# Mitigating the Impact of MR Sequence Parameters: Increasing the Robustness of DL‐Based Cortical Thickness Estimates

**DOI:** 10.1002/hbm.70560

**Published:** 2026-06-07

**Authors:** Timo Blattner, David Romascano, Richard McKinley, Michael Rebsamen, Anke Salmen, Maximilian Pistor, Robert Hoepner, Roland Wiest, Piotr Radojewski, Christian Rummel, Milena Capiglioni

**Affiliations:** ^1^ Support Center for Advanced Neuroimaging (SCAN), University Institute of Diagnostic and Interventional Neuroradiology University of Bern, Inselspital, Bern University Hospital Bern Switzerland; ^2^ Balgrist University Hospital Zurich Switzerland; ^3^ Department of Neurology, Inselspital Bern University Hospital and University of Bern Bern Switzerland; ^4^ Translational Imaging Center (TIC) Swiss Institute for Translational and Entrepreneurial Medicine, Sitem‐Insel Bern Switzerland; ^5^ European Campus Rottal‐Inn Technische Hochschule Deggendorf Pfarrkirchen Germany; ^6^ High‐Field MR Center Max Planck Institute for Biological Cybernetics Tübingen Germany

**Keywords:** brain morphometry, contrast, cortical thickness, deep learning, MR, multiple sclerosis, robustness

## Abstract

Cortical thickness measurements from MRI are increasingly used as biomarkers for neurodegenerative disease progression. However, variations in MRI acquisition parameters, such as inversion time (TI) and repetition time (TR), which are common in clinical settings, can compromise the reliability and sensitivity of these measurements. We fine‐tuned a deep‐learning‐based segmentation tool (DL+DiReCT) to reduce its dependence to image contrast variations by training it on simulated MPRAGE images derived from quantitative relaxation maps. Fine‐tuning markedly reduced contrast sensitivity, with the Pearson correlation coefficient decreasing from −0.644 to 0.094. Evaluation on a synthetic atrophy dataset demonstrated that our model accurately replicated atrophy trends with minimal underestimation, outperforming FreeSurfer and SynthSeg. When applied to a dataset of relapsing–remitting multiple sclerosis (RRMS) patients, the fine‐tuned model showed a substantial reduction in contrast sensitivity and maintained stable performance after controlling for covariates such as age, sex, field strength, and Expanded Disability Status Scale (EDSS) score. Overall, the proposed approach achieves robust contrast invariance without sacrificing sensitivity to cortical atrophy, offering a practical improvement for longitudinal and multi‐center clinical studies.

## Key Points

- Changes in MR acquisition settings, specifically of TI and TR, which are common in the clinical setting, affect WM/GM contrast, which in turn affects cortical thickness measurements.
- Deep learning models can be fine‐tuned on synthetic MRI simulation across different contrasts for more robust cortical thickness measurements.

## Graphical abstract

Left: Our finetuned model reduces dependence on image contrast significantly (top), while conserving sensitivity to synthetic atrophy (bottom). Right: For the finetuning procedure, MRIs with varying grey‐white contrast were synthesized using Deichmann's equations on T1 and PD maps. The segmentation ground truth for all synthetic images was generated by running FreeSurfer on the MRI with highest image contrast. Cortical thickness estimated with various methods was finally compared.

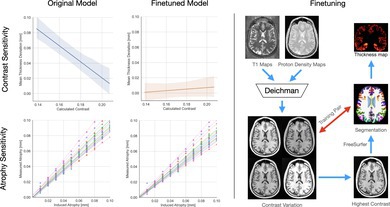

## 1 Introduction

Quantitative brain morphometry, derived from T1‐weighted (T1w) MRI, has become a key imaging biomarker in neuroscience research and is increasingly used to assess neurodegeneration in clinical applications. In multiple sclerosis (MS), the latest no evidence of disease activity (NEDA‐4) criteria include brain atrophy measurement as an imaging biomarker of disease progression (Kappos et al. 2016). Brain volume loss is thought to be accelerated in patients with MS, ranging from 0.5%–1.35% per year in patients with relapsing–remitting MS (RRMS), compared to 0.1%–0.3% in healthy individuals (De Stefano et al. 2014). Periodic MRI scans, typically every 6 months to 1 year, are often a standard part of MS management to monitor disease progression and ensure treatment safety.

Neuro‐morphometric metrics, such as cortical thickness, are typically derived after segmentation of high‐resolution MRI scans. While measurements can be highly reproducible when using consistent imaging protocols (same scanner, sequence, and acquisition parameters) (Han et al. 2006; Hedges et al. 2022; Jovicich et al. 2013), they are sensitive to variations in white matter (WM)/gray matter (GM) tissue contrast. Previous work has shown that several acquisition‐related factors can influence morphometric estimates through their impact on image contrast, including scanner manufacturer and model (Han et al. 2006; Jovicich et al. 2013), head coil (Panman et al. 2019), and RF transmit field (B1) inhomogenities (Duché et al. 2017). In clinical settings, where acquisition parameters such as inversion Time (TI) and repetition time (TR) are often not standardized, these variations can change tissue contrast and introduce significant bias into segmentation, and therefore thickness measurements. For instance, FreeSurfer (Fischl 2012; Fischl and Dale 2000), one of the most widely adopted tools in neuro‐morphometry, has been shown to exhibit bias in response to variations in tissue contrast (Han et al. 2006; Hedges et al. 2022; Kruggel et al. 2010). In a prior study (Rebsamen et al. 2024), we demonstrated that cortical volume and thickness measurements are negatively correlated with WM/GM contrast, irrespective of the methodology used to derive these metrics. This issue also extends to deep learning‐based segmentation tools, such as DL+DiReCT (Rebsamen, McKinley, et al. 2023; Rebsamen et al. 2020), which are trained on FreeSurfer segmentations (prone to the same bias) for training. Although DL+DiReCT has shown enhanced sensitivity in cortical thickness measurement compared to FreeSurfer (Rebsamen et al. 2020; Rusak et al. 2022), it inherits the same tissue contrast sensitivity bias. Consequently, tissue contrast variations can influence cortical thickness estimates by as much as 5%, corresponding to a deviation of 0.1 to 0.2 mm (Rebsamen et al. 2024).

Several recent approaches have been developed to address the issue of contrast sensitivity in neuro‐morphometry, with the aim of improving the robustness of segmentation tools across varying acquisition protocols. Among these, SynthSeg (Billot et al. 2023) uses a domain‐randomization strategy to synthetically generate a variety of MRI images from a segmentation, training their model to segment these synthetically generated images. While promising, SynthSeg relies on fully synthetic images to generalize to a large range of sequences and slice thicknesses, while not necessarily capturing realistic image contrasts. In contrast, (Borges et al. 2019, 2024) propose a physics‐based approach, using multiparameter mapping (MPM) to simulate T1w images from quantitative MRI maps, via signal equations to generate simulated structural 3D images. However, the ground truth in this approach is derived from Gaussian mixture models applied to T1‐weighted images, and does not directly incorporate regional segmentation nor cortical thickness measurements.

In this study, we extend Borges et al.'s approach to mitigate contrast bias inDL+DiReCT. We simulate a range of MPRAGE images from quantitative relaxation maps with varying acquisition parameters, while using FreeSurfer to derive the segmentation at a fixed contrast. The primary objective is to enhance the robustness of DL+DiReCT to acquisition variations, while preserving the accuracy and reliability of FreeSurfer's segmentations. This work aims to improve the reliability and consistency of neuro‐morphometric tools, particularly in the context of longitudinal monitoring and multicenter studies, where inherent variability in acquisition protocols is unavoidable.

## 2 Methods

**FIGURE 1:**
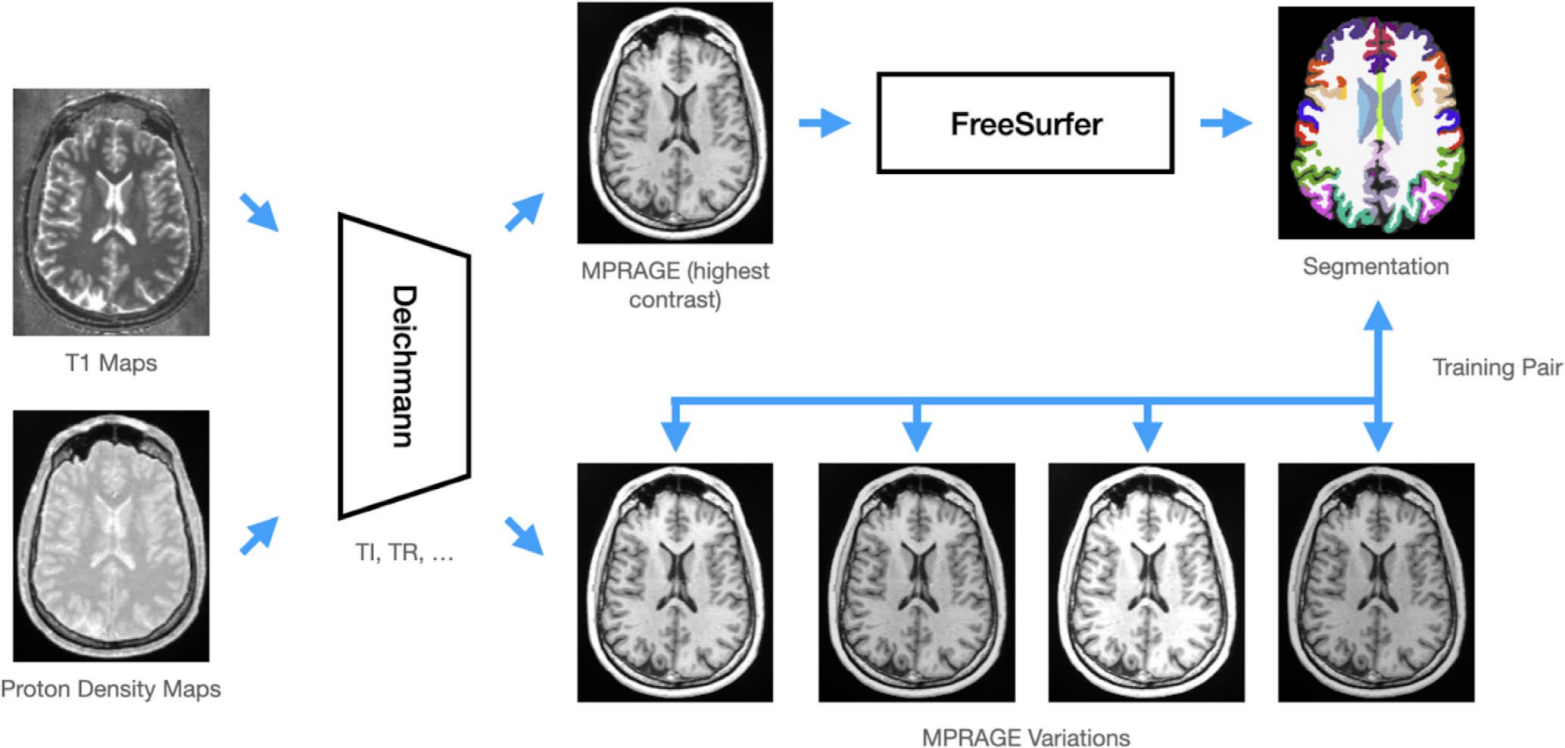
Fine‐tuning method: we synthetically generate MPRAGE images from MPMs with varying sequence parameters and match them to the FreeSurfer segmentation from the highest contrast image.

To enhance robustness against contrast variations in MPRAGE images introduced by changes in TI and TR, we fine‐tuned our original DL+DiReCT model (v6) using a synthetically generated training set. These synthetic images were generated using established signal equations applied to multi‐parametric maps (MPMs), simulating a range of acquisition settings. The images were paired with a single reference segmentation derived from the highest‐contrast to enforce consistency across TI and TR variations. Figure 1 illustrates the overall retraining pipeline.

### 2.1 Study Cohorts

#### 2.1.1 Training Datasets

For fine‐tuning, we used the quantitative T1 and PD maps from Clark and Maguire (2023). The dataset includes *N* = 217 healthy adults (mean age: 29, range 20–41, 109 females, 108 males). The quantitative MPMs were derived using the hMRI‐toolbox (Tabelow et al. 2019).

#### 2.1.2 Evaluation Datasets

##### 2.1.2.1 Phantom of Bern

To assess the impact of image contrast on cortical thickness measurements, we acquired T1w MPRAGE scans from three male subjects (ages 47, 40, and 32) using the ADNI‐3 protocol (Weiner et al. 2017).

##### 2.1.2.2 Atrophy Dataset

To benchmark the sensitivity of our method to cortical atrophy, we used the synthetic dataset from Rusak et al. (Rusak et al. 2022). This dataset consists of T1w images that were synthetically generated using a generative model from partial volume maps, by progressively and globally introducing atrophy at regular intervals. Each of the 20 subjects collected from the ADNI dataset (Weiner et al. 2017), has two atrophy series comprising 10 levels of induced atrophy: a fine‐grade series (0–0.1 mm) and a coarse‐grade series (0–0.1 mm), for the same contrast. For our main analysis, we excluded the coarse‐grade atrophy series because some regions of the cortex become thinner than 1 mm, meaning that for larger levels of induced atrophy, the atrophy would exceed the cortex thickness and be capped at 0 mm, effectively biasing measurements (Rusak et al. 2022). However, results for the coarse atrophy series are still reported in Figure A2.

##### 2.1.2.3 MS

To explore the clinical relevance of our model finetuning, we utilized an in‐house dataset comprising consecutive MRI scans from patients diagnosed with RRMS who were undergoing treatment with natalizumab (Tysabri), a disease‐modifying therapy to reduce relapses. The dataset includes a total of 285 T1w MRI scans (192 from female patients and 93 from male patients), acquired from 64 individuals (mean age at scan time: 36.7 years; age range: 17.1–60 years). Each patient had a mean of 4.83 scans (range: 1–7 scans). Associated with the time point of each scan, the Expanded Disability Status Scale (EDSS) scores were recorded at the time of each scan, with 164 scans from patients with mild disability (0–3.5), 83 with moderate disability (4–5.5), and 38 with severe disability (6–9.5).

### 2.2 MRI Acquisition Protocols

MRI data used in this study originated from both publicly available datasets and in‐house acquisitions. A summary of the main acquisition parameters is provided below; detailed protocol descriptions are available in the corresponding original publications.

#### 2.2.1 Training Datasets

Quantitative T1 and proton density (PD) maps were obtained from the dataset released by Clark and Maguire (2023). Data were acquired on three Siemens Magnetom TIM Trio 3 T scanners equipped with 32‐channel head coils at a single imaging center. MPMs were derived from a 3D multi‐echo fast low‐angle shot (FLASH) sequence at 0.8 mm isotropic resolution. Full acquisition parameters are described in Clark and Maguire (2023).

#### 2.2.2 Phantom of Bern Dataset

The Phantom of Bern dataset was acquired in‐house on a Siemens Prisma 3 T scanner with a 64‐channel head/neck coil during a single imaging session (Rebsamen et al. 2024; Rebsamen, Romascano, et al. 2023). T1w MPRAGE images were obtained following the ADNI‐3 protocol (Weiner et al. 2017). Acquisition parameters common to all scans were: gradient readouts = 208, flip angle = 9°, echo spacing = 7.1 ms, GRAPPA factor/reference lines = 2/24, and voxel size = 1 × 1 × 1 mm^3^. Across scans, TI and TR were systematically varied to modulate image contrast, with nine TI/TR values pair: [(0.8/1.7), (0.9/2), (1.1/2.3), (1.1/2), (0.9/1.7), (1.1/1.84), (0.9/2.3), (0.9/2.3), (1.1/2.6)] s. The lowest‐contrast acquisition used TI=1100 ms and TR=1840 ms, whereas the highest‐contrast acquisition used TI=900 ms and TR=2300 ms. The highest‐contrast scan was repeated at the beginning and end of the session to assess sources of variability unrelated to contrast changes.

#### 2.2.3 Synthetic Atrophy Dataset

The synthetic atrophy dataset from Rusak et al. (2022) was generated from T1w images acquired as part of the ADNI study (Weiner et al. 2017). Original images were collected using standardized ADNI acquisition protocols for 3T scanners. Detailed acquisition parameters are provided in the ADNI documentation and in Rusak et al. (2022).

#### 2.2.4 MS Dataset

The in‐house MS dataset was acquired on Siemens MRI scanners operating at 1.5 T (170 scans in total: 122 Avanto, 30 Aera, 18 Avanto Fit) and 3 T (115 scans in total: 5 TrioTim, 103 Verio, 2 Prisma Fit, 4 Skyra Fit, 1 Vida). TIs ranged from 0.9 to 1.1 s (mean = 1.08), and TRs ranged from 1.5 to 2.53 s (mean = 2.05). A full table with the acquisition parameters for each subject is given in Supporting Information 1.

### 2.3 MRI Preprocessing and Analysis Pipeline

#### 2.3.1 Models

We evaluated mean global cortical thickness estimates over both hemispheres from the original model (*DL+DiReCT* v6), the fine‐tuned model (DL+DiReCT v8), as well as two reference methods: *FreeSurfer* and *SynthSeg* (v2.0) (Billot et al. 2023).

Freesurfer includes dedicated skull‐stripping and intensity normalization procedures. For DL+DiReCT, images are first skull‐stripped using the HD‐BET, then segmented, and DiReCT is used to derive cortical thickness from the segmentation and probability maps (Rebsamen et al. 2020, Das et al. 2009). SynthSeg does not require any prior skull‐stripping, but does not provide cortical thickness by default. Therefore, we used its posterior probability maps and applied the same postprocessing steps as in DL+DiReCT to obtain thickness measurements.

#### 2.3.2 Synthetic Image Generation

Synthetic MPRAGE images with varying contrasts were generated from each subject's PD and T1 maps using Bloch equation‐based simulations described by Deichmann et al. (2000). The acquisition parameters based on the ADNI3 protocol (Weiner et al. 2017) were held constant: gradient readouts = 208, flip angle $=9^{{}^{\circ}}$, echo spacing =7.1ms, GRAPPA factor/reference lines =2/24, and voxel size = $1\times 1\times 1{\mathrm{mm}}^{3}$. Variable TI values ranged from 700 to 1200ms in 100ms steps and TR values ranged from 1600 to 2600ms in 200ms steps. These ranges reflect the variability commonly observed in our clinical population. To better match typical head positioning at our site, simulated MPRAGE images were rotated $=30^{{}^{\circ}}$ around the left–right axis and resampled to 1mm isotropic resolution (Blattner et al. 2025).

#### 2.3.3 Fine‐Tuning

Ground truth labels are obtained using FreeSurfer version 7.3.2 (Fischl 2012) from the highest contrast image, which has the best anatomical delineation. Prior work has shown that DL+DiReCTyields improved segmentation and thickness estimates compared to FreeSurfer, but also suffers from its biases (Rebsamen et al. 2020). Therefore, we seek to enforce consistency across contrast by relying on the highest contrast segmentation as ground truth. DL+DiReCT predicts 101 anatomical labels as defined in (Rebsamen, McKinley, et al. 2023), encompassing white matter, cortical gray matter, subcortical structures, and cortical regions according to the Desikan–Killiany atlas (Desikan et al. 2006). We added an additional label for cerebrospinal fluid (CSF), which allows us to modify our previous threshold‐based cortical parcellation criterion to an argmax‐based labeling rule. In this updated approach, the label corresponding to the highest predicted probability is assigned. DL+DiReCT (Rebsamen et al. 2020), built on a U‐Net architecture (Mckinley et al. 2019; McKinley et al. 2021), was originally trained on 1 mm isotropic, skull‐stripped T1‐weighted images, including contrast‐enhanced scans from MS patients (Rebsamen, McKinley, et al. 2023). To enhance robustness against contrast variability while retaining the model's prior knowledge, we fine‐tuned the original network on our synthetic dataset for an additional 30 epochs, following the initial 100 training epochs.

Each training batch consisted of 16 images from the same anatomical slice simulated under different acquisition settings, paired with the segmentation derived from the highest contrast image $\left(\mathrm{TR}=2300\mathrm{ms},\mathrm{TI}=900\mathrm{ms}\right)$. We combined the focal loss (Lin et al. 2017) with a pairwise Euclidean distance loss to handle class imbalance and encourage consistency across contrast variations. The focal loss

(1) $$L_{\text{focal}}=-\sum_{i=1}^{n}{\left(1-p\right)}^{2}\log p_{i},$$

where $p_{i}$ is the predicted probability for class i, focuses on harder examples by down‐weighting well‐classified ones. The pairwise Euclidean distance loss

(2) $$L_{\text{pairwise}}=\sum_{a=1,b=2,a\neq b}^{n}{\left(p_{a}-p_{b}\right)}^{2}$$

penalizes differences between the predicted probabilities $p_{a}$ and $p_{b}$ across different contrasts, thereby encouraging consistency across different contrast settings. The total loss is a weighted sum of both terms. To further enhance local contrast robustness, we applied random bias field augmentations using the TorchIO library (Pérez‐García et al. 2021).

### 2.4 Evaluation Experiments

#### 2.4.1 Contrast Sensitivity

For each subject and method, we fit a linear regression model with mean cortical thickness as the dependent variable and the synthetic WM/GM tissue contrast (calculated contrast) as the independent variable. Contrast was estimated using the normalized intensity difference:

(3) $$C\text{ontrast}=\frac{|S_{\mathrm{GM}}-S_{\mathrm{WM}}|}{|S_{\mathrm{GM}}+S_{\mathrm{WM}}|}$$

where $S_{GM}$ and $S_{WM}$ represent the MPRAGE signal intensities for gray and white matter, calculated using the Deichmann signal equation (Deichmann et al. 2000). Tissue properties were set to $\rho =85/70\mathrm{kg}/m^{3}$ (Hornak 1996) and T1=1820/1084ms (Stanisz et al. 2005) for the GM and WM, respectively. Pearson's correlation tests were used to evaluate the statistical significance of the observed relationships, both on global mean thickness and regional thickness values from the Desikan–Killiany atlas. To enable comparison across models, cortical thickness values were normalized by subtracting the mean thickness obtained from the two highest‐contrast scans for each subject. Additionally, we evaluated the standard deviation of cortical thickness measurements as a function of contrast for our models and SynthSeg.

#### 2.4.2 Atrophy Sensitivity

We calculated the correlation between the known induced atrophy and the atrophy measured by each model, where measured atrophy is defined as the difference in mean cortical thickness between the un‐atrophied and atrophied images. To assess the model's ability to detect subtle changes, we performed one‐sample *t*‐tests at each atrophy level, testing whether the mean estimated atrophy across subjects was significantly greater than zero. To evaluate measurement accuracy relative to the ground truth, we performed a second set of one‐sample *t*‐tests to determine whether the mean estimated atrophy was significantly smaller than the corresponding induced values, which would indicate underestimation. All measures of atrophies for each level and model passed the Shapiro–Wilk test for normality, justifying the use of a parametric *t*‐test. To account for the correlation of repeated measurements within each subject, we also included the Freesurfer Longitudinal pipeline (Reuter et al. 2012). Subject‐wise templates were created using all subject scans, then each scan was registered to the template, and freesurfer's recon‐all pipeline was applied to the registered scans.

#### 2.4.3 Application to Patients With MS

To assess the influence of image contrast on mean cortical thickness, we used a normative‐modeling approach. We first modeled age‐related cortical thickness variation in healthy controls using polynomial regression as implemented in the ScanOMetrics toolbox (Romascano et al. 2024; Rummel et al. 2018, 2017). The normative dataset comprised 254 scans (155 from female and 99 from male subjects) from 226 healthy subjects acquired in our institution (mean age at scan time: 22.7 years; age range: 6–68 years). We computed the residuals from these age‐predicted values and fitted a linear mixed‐effect model to assess the effects of sex, scanner field strength, scanner ID, synthetic contrast, and EDSS score as covariates. Regional contrast contributions to normative thickness residuals were obtained by fitting the same statistical model to the residual thickness of each of the 68 cortical regions, and correcting the corresponding *p*‐values for multiple comparisons using Bonferroni correction (*p* < 0.05/68 = 0.000735). The effect of EDSS on regional residual thicknesses was also evaluated.

To ensure that lesions on MS scans did not bias thickness estimates and subsequent statistics, the same analysis was repeated on lesion‐filled scans (Uhr et al. 2025). FLAIR scans acquired during the same session as the T1w scans were used to segment lesions by applying NablaNet (McKinley et al. 2016). Lesion masks were aligned with the T1w scan by applying the FLAIR to T1w transformation estimated using FLIRT (Jenkinson and Smith 2001). Our finetuned model was applied to the lesion‐filled scans; normative thickness residuals were computed using our 226 healthy subject normative model, and statistics were computed using the linear mixed‐effect models described above.

## 3 Results

### 3.1 Contrast Sensitivity

**FIGURE 2:**
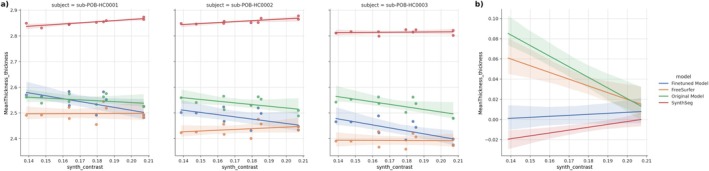
Mean cortical thickness in dependence of the calculated contrast of our Phantom of Bern dataset with each model for (a) all three subjects and (b) grouped by normalization with respect to the highest contrast measures.

In our quantitative phantom dataset, both FreeSurfer and the original DL+DiReCT model exhibited a negative correlation between cortical thickness and image contrast across all three subjects (Figure 2a). The original model showed a statistically significant negative correlation between cortical thickness and contrast in two of the three subjects.

After fine‐tuning, DL+DiReCT showed improved robustness to contrast, with the slope of the regression line diminishing from −1.05 (CI = [−1.57, −0.54]) to 0.1 (CI = [−0.33, 0.53]) (see Figure 2b). Furthermore, the Pearson correlation coefficient also decreased from *r* = −0.644 (CI = [−0.65, −0.64]) to r = 0.10 (CI = [0.08, 0.11]), indicating that the relationship between cortical thickness and contrast was significantly weakened, with no statistically significant correlation in any of the subjects.

**FIGURE A1:**
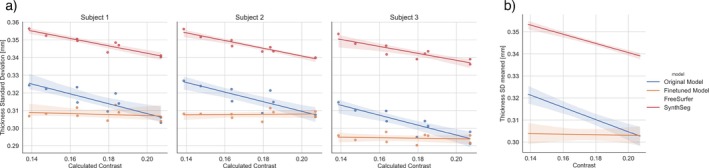
Standard deviation of cortical thickness measurement in dependence of the calculated contrast of our Phantom of Bern dataset for (a) all three subjects and (b) grouped by normalization with respect to the highest contrast measures.

By comparison, SynthSeg consistently reported about 10% higher mean cortical thickness values than FreeSurfer and either version of our model, and showed the lowest variance across all models. SynthSeg also shows a strong positive correlation with contrast,statistically significant in two subjects (Table A1, Appendix A). The standard deviation of cortical thickness measurements is reported in Figure A1 (Appendix A1).

**FIGURE 3:**
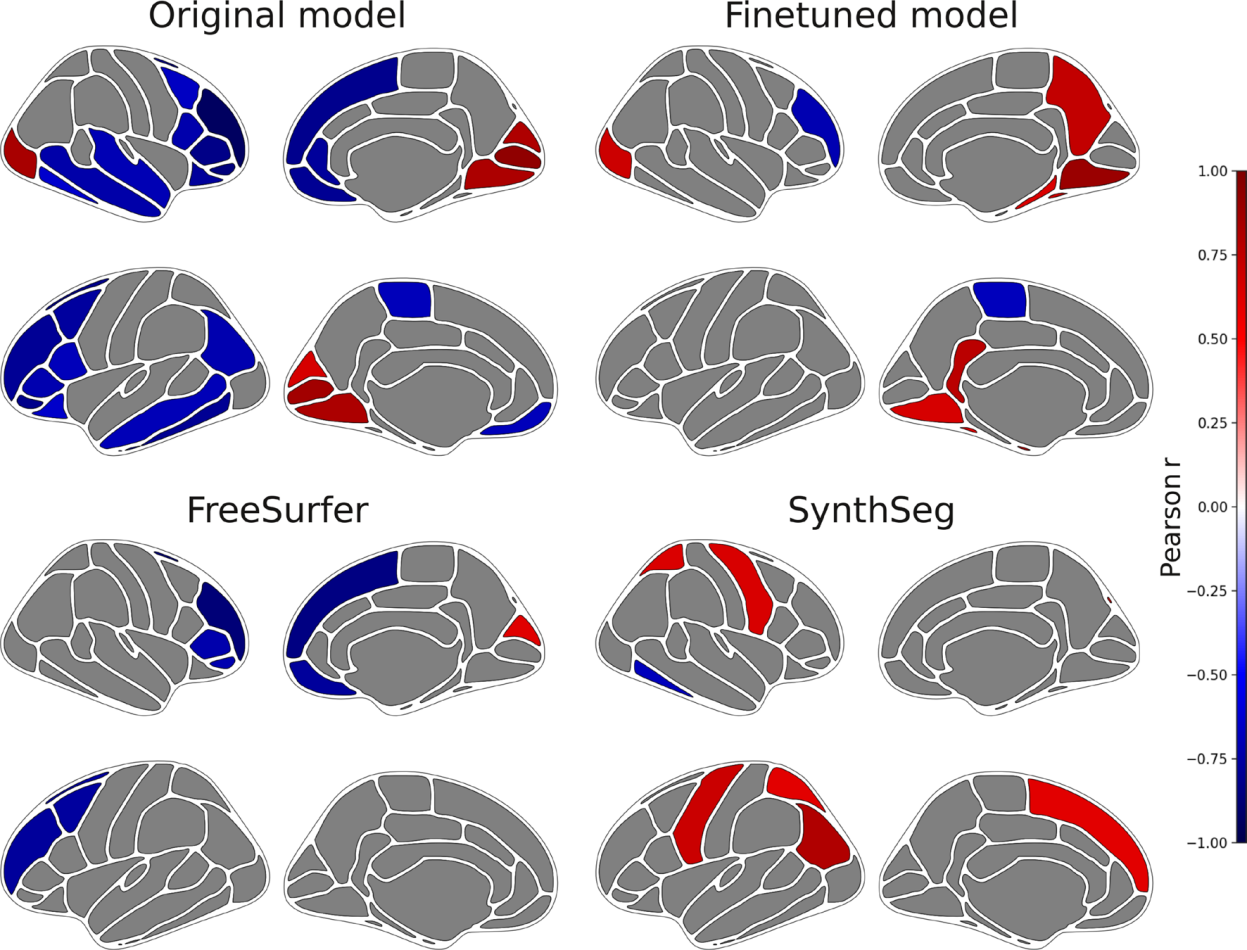
Phantom of Bern: Pearson's correlation (*r*) for regions showing significant associations (*p* < 0.000735) between regional cortical thickness and calculated contrast for all our models, normalized across subjects.

Figure 3 shows the regional correlation between calculated contrast and local cortical thickness across subjects. The original model revealed statistically significant associations (p<0.05/68) in several frontal and temporal regions, with predominantly negative correlations; positive correlations were observed in occipital regions. Overall, 34 out of the 68 regions exhibited significant correlations. In comparison, the fine‐tuned model showed a reduction in the number of significant regions, with only 13 regions remaining significant. These regions were predominantly located in the parietal and occipital lobes. Notably, the fine‐tuned model showed a marked decrease in the absolute Pearson correlation coefficients across the majority of regions, with 51 out of 68 regions exhibiting smaller absolute values. The mean absolute correlation coefficient decreased from 0.55 in the original model to 0.35 in the fine‐tuned model. FreeSurfer showed a pattern similar to that of the original model, with 11 regions in the frontal lobe showing predominantly negative correlations. SynthSeg, on the other hand, displayed a different pattern, with positive correlations predominantly in the frontal and parietal lobes. However, only seven regions reached statistical significance in SynthSeg.

**FIGURE 4:**
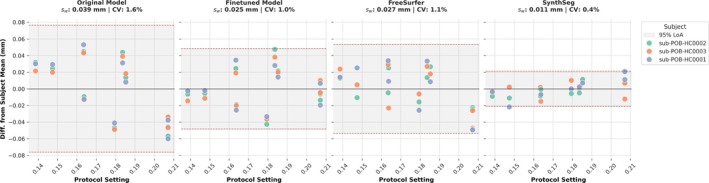
Robustness of cortical thickness estimates across protocol variations. Intrasubject repeatability was assessed using Bland–Altman analysis for repeated measures. Shaded areas represent the 95% limits of agreement (±1.96 × *s*_w_) derived from within‐subject variance.

To evaluate the robustness of the method against protocol variability, we performed a Bland–Altman analysis for repeated measures. Within‐subject standard deviation ($s_{w}$) was calculated for each method using a one‐way analysis of variance (ANOVA) to partition measurement error from inter‐subject biological variance. Robustness was quantified via 95% limits of agreement ($\pm 1.96\;s_{w}$), the repeatability coefficient ($\mathrm{RC}=2.77\;s_{w}$), and the within‐subject coefficient of variation (CV). This approach assesses the stability of each method by measuring the dispersion of residuals, defined as the difference between individual protocol measurements and the subject's mean, across the eight imaging configurations (Figure 4).

### 3.2 Atrophy Sensitivity

**FIGURE 5:**
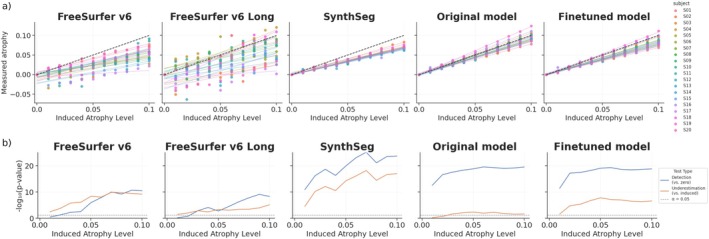
Synthetically induced atrophy (0.0–0.1 mm) versus (a) the mean measured atrophy of each model for 20 subjects and (b) the atrophy detection in blue (should be large) and underestimation of atrophy in yellow (should be small), the dotted line corresponds to the α=0.05 significance level (uncorrected for multiple comparisons).

All models showed a statistically significant linear correlation with the level of induced atrophy (Figure 5a). The original model best reproduced the expected trend, with a slope of 0.94 (CI = [0.92, 0.97]), closely matching the ground truth. The fine‐tuned model showed a slightly weaker but still strong slope of 0.83 (CI = [0.80, 0.85]), outperforming Freesurfer longitudinal (slope 0.72, CI = [0.61, 0.82]), FreeSurfer cross‐sectional (slope 0.57, CI = [0.51, 0.63]) and SynthSeg (slope 0.70, CI = [0.68, 0.71]) in capturing the progression of atrophy.

**FIGURE A2:**
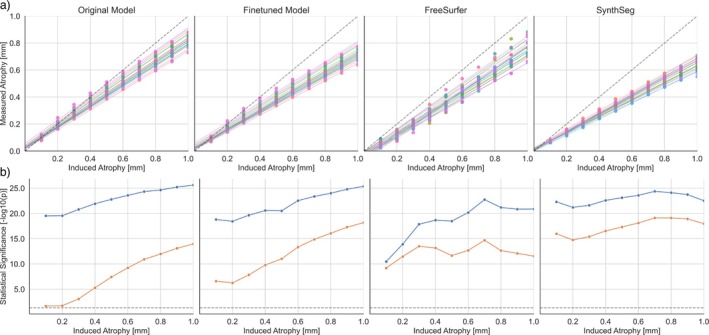
Synthetically induced atrophy (0.0–1.0 mm) versus (a) the mean measured atrophy of each model for 20 subjects and (b) the atrophy detection in blue (should be large) and underestimation of atrophy in yellow (should be small), the dotted line corresponds to the α=0.05 significance level.

Figure 5b shows each method's ability to *detect* synthetically induced atrophy and it's tendency to *underestimate* the extent of the atrophy. Ideally, the values for detection should be large, whereas those for underestimation should be small. All models were able to detect atrophy across the full range of induced atrophy levels, although FreeSurfer's detection were less significant due to it's higher variance (*detection* in Figure 5b). All models tend to underestimate atrophy for values exceeding 0.02 mm (*underestimate* in Figure 5b). For the original DL+DiReCT model, detected and induced atrophy values are closest, whereas the fine‐tuned model slightly underestimates atrophy. Further analysis of larger atrophy values, ranging from 0.1 to 1 mm, is provided in Figure A2.

### 3.3 Application to Patients With MS

**FIGURE 6:**
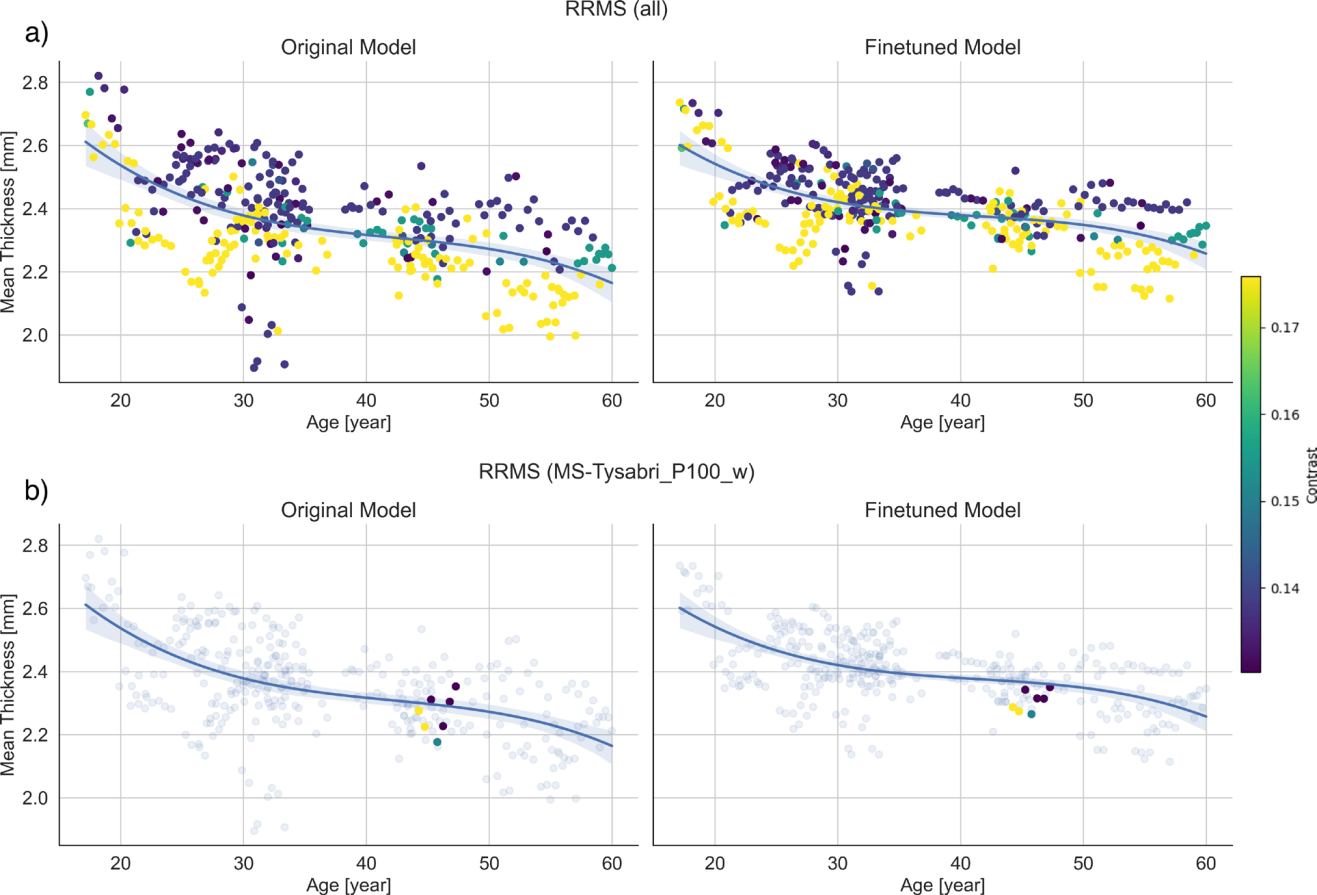
Mean cortical thickness as a function of age, (a) for all MS patients and (b) for a representative patient scanned with different parameters, for our original model (left) and fine‐tuned model (right). Color indicates WM/GM calculated contrast.

Figure 6a shows the relationship between age and mean cortical thickness across all RRMS scans for both the original and fine‐tuned DL+DiReCT models. Higher image contrast generally yielded lower thickness estimates. The fine‐tuned model showed a reduction in variance (variance = 0.0086) relative to the original model (variance = 0.017), together with a decrease in the number of outliers, indicating improved robustness to contrast variations. Figure 6b similarly shows reduced contrast dependence in a single patient scanned under varying contrast conditions.

**FIGURE 7:**
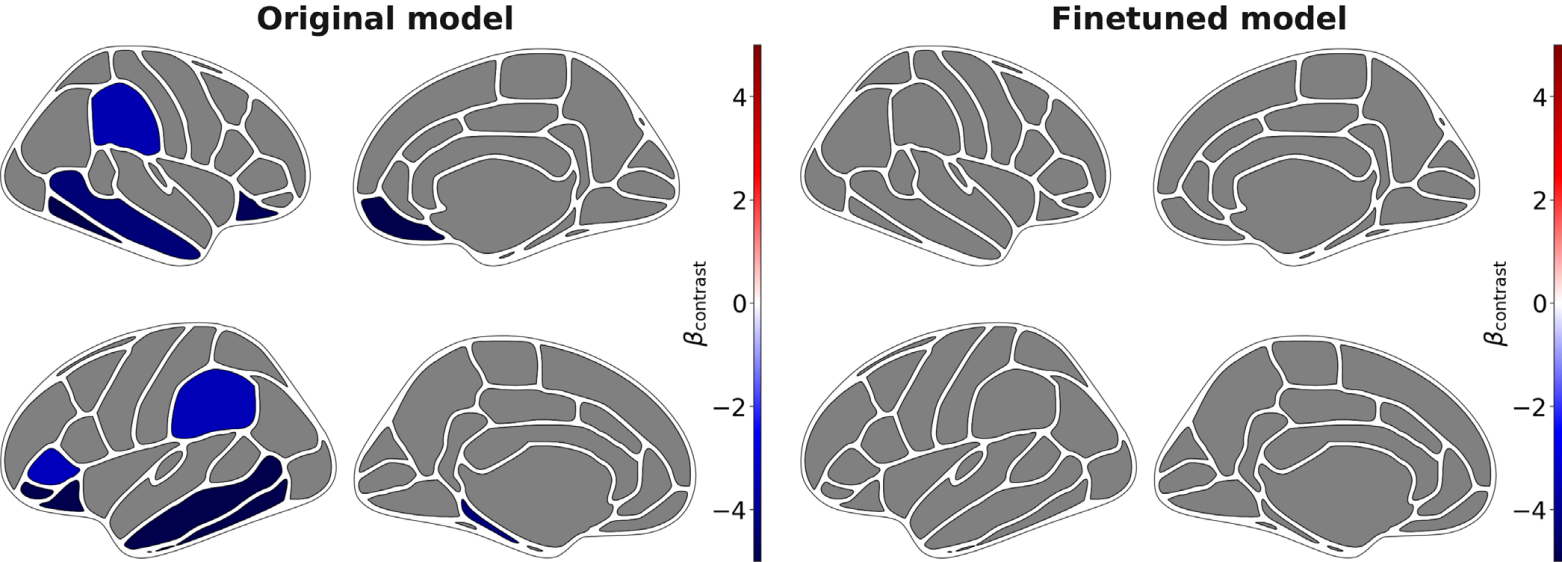
Regional map of $\beta_{\text{contrast}}$, for the original (v6) and finetuned (v8) DL + DiReCT models. Regions that did not survive Bonferroni correction (*p* ≥ 0.000735) are masked in gray. Nonthresholded regional maps are available in Supporting Information 3.

Fitting a linear mixed effect model on the residuals of mean cortical thickness in the RRMS dataset revealed a significant negative effect of contrast in the original model ($\beta_{\text{contrast}}=-2.155$, *p* = 0.003, CI = [−3.582, −0.728]). In the fine‐tuned model, this effect was markedly reduced and no longer significant ($\beta_{\text{contrast}}=-0.603$, *p* = 0.171, CI = [−1.467, 0.261]), indicating improved robustness to contrast variation in clinical data. Significant regional contributions of $\beta_{\text{contrast}}$ to normative thickness residuals are shown in Figure 7 (*p* < 0.05/68).

Sex and EDSS scores did not significantly contribute to mean cortical thickness residues in either models. Coefficients, *p*‐values and 95% confidence intervals were similar in both models. The fine tuned model lead to $\beta_{\mathrm{sex}}=-0.026$ (*p* = 0.270, CI = [−0.072, 0.020]) and $\beta_{\text{EDSS}}=0.005$ (*p* = 0.128, CI = [−0.002, 0.012]), while the original model led to $\beta_{\mathrm{sex}}=-0.034$ (*p* = 0.272, CI = [−0.093, 0.026]) and $\beta_{\text{EDSS}}=0.002$ (*p* = 0.726, CI = [−0.009, 0.012]). The fine‐tuned segmentation model led to $\sigma_{\mathrm{res}}^{2}=0.001$, $\sigma_{\mathrm{rand}}^{2}=0.007$, AIC = −1038.64, BIC = −987.50 and Log‐Likelihood = 533.32. The original segmentation model led to $\sigma_{\mathrm{res}}^{2}=0.002$, $\sigma_{\mathrm{rand}}^{2}=0.011$, AIC = −778.83, BIC = −727.70, and Log‐Likelihood = 403.42. Full statistical details are provided in the Supporting Information 2. Regarding the regional analysis, EDSS scores did not significantly affect any regional thickness residuals.

Similar results were obtained when using lesion‐filled scans. Mean thickness residuals before and after lesion‐filling showed a pearson correlation coefficient of 0.996 (*p* < 10^−12^). Supporting Information 3 provides example T1w scans before and after lesion filling, regional maps of $\beta_{\text{contrast}}$ obtained from the mixed‐effect model, and full statistical details regarding the normative residuals of the mean thickness.

## 4 Discussion

Variation in MRI acquisition settings, particularly TI and TR, affects WM/GM contrast and have been shown to influence neuro‐morphometric measurements by amounts larger than the changes observed in the early stages of disease (Haller et al. 2016; Rebsamen et al. 2024). In this study, we fine‐tuned our segmentation model (DL+DiReCT) to improve the robustness of cortical thickness estimates to contrast variations induced by changes in TI and TR, using physics‐based simulations of MPRAGE sequences.

After fine‐tuning, we find a marked reduction in the model's dependence on tissue contrast. The Pearson correlation coefficient decreased in magnitude from −0.644 to 0.094 on our real‐world contrast benchmarking dataset and became statistically non‐significant. The number of regions exhibiting significant correlations decreased from 34 to only 13 after fine‐tuning. When comparing the maximal absolute change in cortical thickness, it decreased marginally from 4.2% for the original to 3.2% for the fine‐tuned DL+DiReCT model. For comparison, within‐session repeatability of the original and fine‐tuned models led to much smaller variabilities, that is, only 0.5% change between repeated acquisitions with identical parameters. Additionally, FreeSurfer and SynthSeg exhibited absolute cortical thickness changes of 2.8% and 1.2%, respectively. These results indicate that, although our method demonstrates enhanced stability with respect to TI/TR‐induced contrast variability, it still exhibits slightly higher overall variability compared to FreeSurfer and SynthSeg. However, it is important to note that trends may vary locally across regions.

Our experiment using a synthetic atrophy dataset demonstrates that while our model accurately reproduces the general atrophy trend, it slightly underestimates atrophy when compared to the base models. In contrast, FreeSurfer and SynthSeg fail to replicate the trend with comparable accuracy. Atrophy detection remains high across all models and atrophy levels, with FreeSurfer showing the lowest performance due to higher variance. These results suggest that our method maintains robust contrast invariance without a significant loss in sensitivity for atrophy detection, whereas SynthSeg exhibits lower variance but also reduced sensitivity. Interestingly, SynthSeg consistently reports higher cortical thicknesses, approximately 10% greater than those produced by FreeSurfer and DL+DiReCT, while it is important to note that DL+DiReCT was trained using FreeSurfer segmentations as ground truth. Additionally, we find that the standard deviation of cortical thickness measurements is higher for SynthSeg but decreases as a function of contrast (Figure A1). Visual inspection reveals that SynthSeg tends to merge smaller sulci more frequently, which likely contributes to the observed increase in cortical thickness measurements.

Finally, when applied to our in‐house dataset of RRMS patients,the fine‐tuned model showed a substantial reduction in contrast sensitivity of mean cortical thickness. After controlling for age, the model's dependence on contrast was substantially reduced (linear coefficient from −2.155 to −0.603), and the correlation became non‐significant. In both models, sex, field strength, and EDSS did not significantly contribute to mean thickness residuals. This is consistent with findings by Narayana et al. (Narayana et al. 2013), who observed only modest (even nonsignificant) correlation between global cortical thickness, EDSS, and field strength. While Narayana's et al. regional analysis showed significant correlation between certain cortical thicknesses and EDSS scores, no region survived Bonferroni correction in our analysis. This discrepancy may reflect differences in medication effects between Tysabri, used in our cohort, and the medication used in the CombiRx Trial. A detailed study on morphometry and MS pathophysiology is however beyond the scope of this study; future studies should address the influence of lesion load and medication in morphometric measures.

From a practical perspective, reducing contrast sensitivity may be particularly relevant for longitudinal and multi‐center studies, where protocol differences between sites or over time can introduce systematic biases that confound biological interpretation. In such settings, approaches that improve the robustness of automated morphometry pipelines could complement existing harmonisation strategies, such as protocol standardisation or statistical batch‐correction methods, by reducing contrast‐driven variability at the level of the segmentation model itself.

### 4.1 Limitations

The dataset used for fine‐tuning was limited to a narrow age range (20–40 years), which may have biased the model toward healthier, less atrophied brains. Manual inspection of the fine‐tuned model revealed mis‐segmentations of MS lesions, labeling them as part of the cortex. While MS patients were included in the original training set, the fine‐tuning dataset did not contain MS patients with lesions. Given that cortical thickness measurements are derived by averaging the thickness map across the entire cortex, the impact of these mis‐segmented voxels should be minimal. This was confirmed by evaluating the performance of the fine‐tuned model on lesion‐filled scans (Supporting Information 3). To further enhance model robustness, future studies should aim to include MS patients in the fine‐tuning dataset as well as a broader age range. Unfortunately, quantitative relaxation maps for MS patients are rare; thus, training on a combination of real and synthetic data may be a promising approach to mitigate this problem.

Our study was specifically focused on mitigating the effects of changes in TI and TR, which have a pronounced impact on image contrast. However, other acquisition‐related variations, such as MRI acquisition acceleration techniques (Dieckmeyer et al. 2021) or different scanners (Han et al. 2006), can also introduce additional sources of measurement variability. Althought we inject artificial bias fields during training to increase robustness, we did not explicitly model these effects, even though our results (Figure 3) suggest that some of them might be spatially localized.

Beyond technical factors, physiological variables such as time of day (Trefler et al. 2016; Alfaro‐Almagro et al. 2021; Walters et al. 2001), head‐tilt (Hedges et al. 2022), head motion during scanning (Alexander‐Bloch et al. 2016; Reuter et al. 2015), or hydration level (Duning et al. 2005), may also introduce confounding effects that impact repeatability and, consequently, sensitivity. These variables are often beyond control in clinical settings, contributing to inherent variability. In addition, pathological tissue changes such as edema or inflammation, particularly in MS, can alter WM/GM contrast and influence morphometric measurements. The present work did not explicitly investigate these physiological factors, which remain an important direction for future research, particularly in pathological cohorts.

### 4.2 Outlook

In principle, the simulation framework could be extended beyond TI/TR variations. Our approach relies on closed‐form Bloch equation‐based signal models for the MPRAGE steady state, and analogous formulations exist for MP2RAGE, MDEFT, T2‐weighted, and FLAIR sequences. Because these share the same dependence on quantitative tissue maps (PD, T1, and where relevant T2), extending the pipeline would primarily require substituting the appropriate signal equation. Related efforts have been made to benchmark morphometric tools via T1w simulations (Posselt et al. 2024). Similarly, future work could simulate variations in flip angle, RF transmit field (B1) inhomogeneity, or parallel imaging acceleration factors, allowing the model to learn robustness to a wider range of acquisition‐dependent on local and global intensity variations.

Existing literature suggests that neuro‐morphometric measurements derived from the same sequence and scanner system are generally repeatable across centers (George et al. 2020; Jovicich et al. 2013). Given that sequence parameters rarely match across institutions, an approach like ours, focused on improving robustness to sequence‐related variations, is relevant not only for analyzing heterogeneous data in clinical routine settings, but could also facilitate the pooling of multi‐center datasets, enabling larger‐scale studies.

## Author Contributions

**Timo Blattner:** data curation, methodology, software, formal analysis, investigation, writing – original draft. **David Romascano:** formal analysis, investigation, software, writing – review and editing. **Richard McKinley:** methodology, writing – review and editing. **Michael Rebsamen:** resources, software, writing – review and editing. **Anke Salmen:** data curation, writing – review and editing. **Maximilian Pistor:** data curation, writing – review and editing. **Robert Hoepner:** data curation, writing – review and editing. **Roland Wiest:** funding acquisition, project administration, writing – review and editing. **Piotr Radojewski:** conceptualization, writing – review and editing. **Christian Rummel:** conceptualization, methodology, funding acquisition, writing – original draft. **Milena Capiglioni:** supervision, methodology, investigation, writing – original draft.

## Funding

This work was supported by the Schweizerischer Nationalfonds zur Förderung der Wissenschaftlichen Forschung (204593).

## Ethics Statement

The MS patients dataset was enrolled in the neuroimmunological registry from Inselspital, approved by the Ethikkommision Kanton Bern (KEK‐BE 2017‐01369, KEK‐BE 2016‐02035).

## Conflicts of Interest

Maximilian Pistor received a research grant from the Swiss MS‐Society and travel funding from Alexion and Roche, both unrelated to this work. The other authors declare no conflicts of interest.

## Supporting information


**Table S1:** Acquisition protocol of MS‐Tysabri. Manufacturer = Siemens, Slice thickness = 1 mm, Base resolution = 256, Sequence = GR_IR.
**Figure S1:** Original T1, lesion mask, and resulting lesion‐filled scan for a random MS subject.
**Figure S2:** Regional map of *β*_contrast_ derived from the original model applied on original T1 scans (DL+DiReCT v1), the finetuned model applied to original T1 scans (DL+DiReCT v8), and the finetuned model applied to lesion‐filledscans (DL+DiReCT v8 with lesion‐filling).

## Data Availability

Training dataset: this training dataset is publicly available (Clark and Maguire 2023). Contrast sensitivity: this evaluation dataset is publicly available (Rebsamen, Romascano, et al. 2023). Atrophy sensitivity: this evaluation dataset is publicly available (Rusak et al. 2021). MS evaluation: this evaluation dataset is not publicly available.

## Appendix A

**TABLE A1:** Raw metrics for section contrast sensitivity (Section 3.1), for each model, subject and grouped subject through normalization.

Model	Subject	Slope [CI]	*r* [CI]	*p*	SD	Percentage
Fine‐tuned Model	Sub‐POB‐HC0001	0.026 [−0.98, 1.03]	0.023 [−0.00, 0.05]	0.953	0.025	3.673
	Sub‐POB‐HC0002	0.303 [−0.53, 1.14]	0.310 [0.29, 0.33]	0.417	0.022	3.110
	Sub‐POB‐HC0003	−0.031 [−0.90, 0.84]	−0.032 [−0.06, −0.01]	0.935	0.022	2.879
	Grouped	*0.099 [−0.79, 0.99]*	*0.046 [0.03, 0.06]*	*0.820*	*0.049*	*7.874*
FreeSurfer	Sub‐POB‐HC0001	−0.347 [−1.02, 0.33]	−0.417 [−0.44, −0.40]	0.264	0.019	2.029
	Sub‐POB‐HC0002	−0.661 [−1.49, 0.17]	−0.580 [−0.60, −0.56]	0.101	0.026	3.076
	Sub‐POB‐HC0003	−0.995 [−1.84, −0.15]	−0.724 [−0.74, −0.71]	**0.028**	0.031	3.356
	Grouped	*−0.668 [−1.08, −0.26]*	*−0.557 [−0.57, −0.55]*	***0.003***	*0.027*	*3.903*
Original model	Sub‐POB‐HC0001	−1.136 [−2.27, −0.00]	−0.667 [−0.68, −0.65]	**0.050**	0.039	4.099
	Sub‐POB‐HC0002	−0.861 [−1.97, 0.25]	−0.571 [−0.59, −0.55]	0.108	0.034	3.787
	Sub‐POB‐HC0003	−1.160 [−2.20, −0.12]	−0.704 [−0.72, −0.69]	**0.034**	0.037	4.760
	Grouped	*−1.052 [−1.97, −0.13]*	*−0.427 [−0.44, −0.42]*	***0.026***	*0.056*	*8.739*
SynthSeg	Sub‐POB‐HC0001	0.440 [0.20, 0.68]	0.854 [0.85, 0.86]	**0.003**	0.012	1.501
	Sub‐POB‐HC0002	0.371 [0.13, 0.61]	0.813 [0.80, 0.82]	**0.008**	0.010	1.121
	Sub‐POB‐HC0003	0.041 [−0.29, 0.37]	0.110 [0.08, 0.14]	0.778	0.008	0.901
	Grouped	*0.284 [−0.10, 0.66]*	*0.294 [0.28, 0.31]*	*0.137*	*0.022*	*2.820*

*Note:* Namely, the slope, Pearson's correlation coefficient (*r*) and corresponding *p*‐value of the mean cortical thickness measurement with respect to contrast, and the standard deviation and mean percentage difference in thickness values. In bold, we indicate significant values, in italic the grouped values.
