# Supplementary material for: Mitigating the Impact of MR Sequence Parameters: Increasing the Robustness of DL‐Based Cortical Thickness Estimates
Source: Hum Brain Mapp. 2026 Jun 7;47(8):e70560. doi: 10.1002/hbm.70560 (PMC13243191; doi:10.1002/hbm.70560)
Supplement: Supplementary file 1 — Table S1: Acquisition protocol of MS‐Tysabri. Manufacturer = Siemens, Slice thickness = 1 mm, Base resolution = 256, Sequence = GR_IR. Figure S1: Original T1, lesion mask, and resulting lesion‐filled scan for a random MS subject. Figure S2: Regional map of β contrast derived from the original model applied on original T1 scans (DL+DiReCT v1), the finetuned model applied to original T1 scans (DL+DiReCT v8), and the finetuned model applied to lesion‐filledscans (DL+DiReCT v8 with lesion‐filling). [file HBM-47-e70560-s001.pdf]

Supplementary Material

for the manuscript:

Mitigating the Impact of MR Sequence Parameters: Increasing the Robustness of DL-based Cortical Thickness Estimates

1 Supplementary Table S1

The following table presents the control sequence parameters for each subject within the Multiple Sclerosis Dataset.

Acquisition protocol of MS-Tysabri. Manufacturer = Siemens, Slice thickness = 1mm, Base resolution = 256, Sequence = GR_IR

SUBJECT_ID	Sex	Age	Field Strenght	Model	TE	TR	TI	FA	Partial Fourier	Phase Res.	Receive Coil	% Phase FOV	Acq. Matrix PE	Pixel BW	Phase Overs.	Synth contrast
P071_0	F	44	3	Verio	0.00296	2.53	1.1	7	0.875	0.96	NeckMatrix	87.5	215	130		0.17584
P071_402	F	45	3	Verio	0.00296	2.53	1.1	7	0.875	0.96	HeadMatrix	87.5	215	130		0.17584
P071_590	F	45	3	Verio	0.00201	1.5	0.9	9	1	1	32Ch_Head	100	256	238		0.14921
P071_758	F	46	3	Verio	0.00296	2.53	1.1	7	0.875	0.96	HeadMatrix	87.5	215	130		0.17584
P071_919	F	46	1.5	Avanto	0.00292	1.83	1.1	15	1	1	32Ch_Head	100	256	130	0.25	0.13807
P071_1118	F	47	1.5	Avanto	0.00292	1.83	1.1	15	1	1	32Ch_Head	100	256	130	0.25	0.13807
P075_0	F	31	3	Verio	0.00296	2.53	1.1	7	0.875	0.96	HeadMatrix	87.5	215	130		0.17584
P075_180	F	31	3	Verio	0.00296	2.53	1.1	7	0.875	0.96	HeadMatrix	87.5	215	130		0.17584
P075_356	F	32	3	Verio	0.00296	2.53	1.1	7	0.875	0.96	HeadMatrix	87.5	215	130		0.17584
P075_553	F	32	1.5	Avanto	0.00292	1.83	1.1	15	1	1	32Ch_Head	100	256	130	0.25	0.13807
P076_0	M	32	1.5	Avanto	0.00292	1.9	1.1	15	1	1	32Ch_Head	100	256	130	0.25	0.14193
P076_182	M	33	1.5	Avanto	0.00292	1.9	1.1	15	1	1	32Ch_Head	100	256	130	0.25	0.14193
P076_366	M	33	1.5	Avanto	0.00292	1.83	1.1	15	1	1	32Ch_Head	100	256	130	0.25	0.13807
P076_576	M	34	1.5	Avanto	0.00292	1.83	1.1	15	1	1	32Ch_Head	100	256	130	0.25	0.13807
P076_770	M	34	1.5	Avanto	0.00292	1.83	1.1	15	1	1	32Ch_Head	100	256	130	0.25	0.13807
P076_875	M	35	1.5	Avanto	0.00292	1.83	1.1	15	1	1	32Ch_Head	100	256	130	0.25	0.13807
P086_0	M	31	1.5	Avanto	0.00292	1.9	1.1	15	1	1	32Ch_Head	100	256	130	0.25	0.14193
P086_166	M	32	1.5	Avanto	0.00292	1.9	1.1	15	1	1	32Ch_Head	100	256	130	0.25	0.14193
P086_355	M	32	1.5	Avanto	0.00292	1.72	1.1	15	1	1	HeadMatrix	100	256	130	0.25	0.13201
P086_511	M	33	1.5	Avanto	0.00292	1.72	1.1	15	1	1	32Ch_Head	100	256	130	0.25	0.13201
P086_706	M	33	1.5	Aera	0.00267	1.58	0.9	8	1	1	HeadNeck_20	100	256	150		0.15488
P092_0	F	43	1.5	Avanto	0.00292	1.9	1.1	15	1	1	32Ch_Head	100	256	130	0.25	0.14193
P092_155	F	43	1.5	Avanto	0.00292	1.83	1.1	15	1	1	32Ch_Head	100	256	130	0.25	0.13807
P092_393	F	44	1.5	Avanto	0.00292	1.83	1.1	15	1	1	32Ch_Head	100	256	130	0.25	0.13807
P092_540	F	44	1.5	Avanto	0.00292	1.83	1.1	15	1	1	32Ch_Head	100	256	130	0.25	0.13807
P092_729	F	45	1.5	Avanto	0.00292	1.83	1.1	15	1	1	32Ch_Head	100	256	130	0.25	0.13807
P092_897	F	45	1.5	Avanto	0.00292	1.72	1.1	15	1	1	NeckMatrix	100	256	130	0.25	0.13201
P097_0	F	28	3	Verio	0.00296	2.53	1.1	7	0.875	0.96	HeadMatrix	87.5	215	130		0.17584
P097_177	F	28	3	Verio	0.00296	2.53	1.1	7	0.875	0.96	HeadMatrix	87.5	215	130		0.17584
P097_295	F	29	3	Verio	0.00296	2.53	1.1	7	0.875	0.96	NeckMatrix	87.5	215	130		0.17584
P097_558	F	29	3	Verio	0.00296	2.53	1.1	7	0.875	0.96	HeadMatrix	87.5	215	130		0.17584
P100_0	F	44	3	TrioTim	0.0022	2.53	1.1	9	1	1	HeadMatrix	100	256	200		0.17584
P100_182	F	45	3	TrioTim	0.0022	2.53	1.1	9	1	1	HeadMatrix	100	256	200		0.17584
P100_371	F	45	1.5	Avanto	0.00292	1.83	1.1	15	1	1	32Ch_Head	100	256	130	0.25	0.13807
P100_554	F	46	1.5	Aera	0.00267	1.58	0.9	8	1	1	HeadNeck_20	100	256	150		0.15488
P100_723	F	46	1.5	Avanto	0.00292	1.83	1.1	15	1	1	32Ch_Head	100	256	130	0.25	0.13807
P100_924	F	47	1.5	Avanto	0.00292	1.83	1.1	15	1	1	32Ch_Head	100	256	130	0.25	0.13807
P100_1103	F	47	1.5	Avanto	0.00292	1.83	1.1	15	1	1	32Ch_Head	100	256	130	0.25	0.13807
P103_0	F	35	3	Verio	0.00201	1.5	0.9	9	1	1	32Ch_Head	100	256	238		0.14921
P103_365	F	36	3	Verio	0.00296	2.53	1.1	7	0.875	0.96	HeadMatrix	100	246	130		0.17584
P103_544	F	36	3	Verio	0.00296	2.53	1.1	7	0.875	0.96	HeadMatrix	100	246	130		0.17584
P103_695	F	37	3	Verio	0.00296	2.53	1.1	7	0.875	0.96	32Ch_Head	100	246	130		0.17584
P110_0	F	49	1.5	Avanto	0.00292	1.83	1.1	15	1	1	32Ch_Head	100	256	130	0.25	0.13807
P110_163	F	50	1.5	Avanto	0.00292	1.83	1.1	15	1	1	32Ch_Head	100	256	130	0.25	0.13807
P110_350	F	50	1.5	Avanto	0.00292	1.83	1.1	15	1	1	32Ch_Head	100	256	130	0.25	0.13807
P110_537	F	51	1.5	Avanto	0.00292	1.83	1.1	15	1	1	32Ch_Head	100	256	130	0.25	0.13807
P110_730	F	51	1.5	Aera	0.00267	1.58	0.9	8	1	1	HeadNeck_20	100	256	150		0.15488
P110_879	F	52	1.5	Avanto	0.00292	1.83	1.1	15	1	1	32Ch_Head	100	256	130	0.25	0.13807
P110_1100	F	52	1.5	Avanto	0.00292	1.72	1.1	15	1	1	32Ch_Head	100	256	130	0.25	0.13201
P111_0	F	21	3	Verio	0.00296	2.53	1.1	7	0.875	0.96	32Ch_Head	87.5	215	130		0.17584
P111_195	F	22	3	Verio	0.00296	2.53	1.1	7	0.875	0.96	32Ch_Head	87.5	215	130		0.17584
P111_414	F	23	3	Verio	0.00296	2.53	1.1	7	0.875	0.96	NeckMatrix	87.5	215	130		0.17584
P111_608	F	23	3	Verio	0.00296	2.53	1.1	7	0.875	0.96	HeadMatrix	87.5	215	130		0.17584
P111_788	F	24	3	Verio	0.00296	2.53	1.1	7	0.875	0.96	32Ch_Head	100	246	130		0.17584
P111_973	F	24	3	Verio	0.00296	2.53	1.1	7	0.875	0.96	HeadMatrix	100	246	130		0.17584
P113_0	F	27	3	Verio	0.00296	2.53	1.1	7	0.875	0.96	NeckMatrix	87.5	215	130		0.17584
P113_218	F	28	3	Verio	0.00296	2.53	1.1	7	0.875	0.96	HeadMatrix	87.5	215	130		0.17584
P113_413	F	28	3	Verio	0.00296	2.53	1.1	7	0.875	0.96	HeadMatrix	87.5	215	130		0.17584
P113_589	F	29	3	Verio	0.00296	2.53	1.1	7	0.875	0.96	HeadMatrix	87.5	215	130		0.17584
P114_0	F	45	3	Verio	0.00296	2.53	1.1	7	0.875	0.96	32Ch_Head	87.5	215	130		0.17584
P114_209	F	46	3	Verio	0.00296	2.53	1.1	7	0.875	0.96	HeadMatrix	87.5	215	130		0.17584
P114_399	F	46	3	Verio	0.00296	2.53	1.1	7	0.875	0.96	HeadMatrix	87.5	215	130		0.17584
P114_558	F	47	3	Verio	0.00296	2.53	1.1	7	0.875	0.96	32Ch_Head	87.5	215	130		0.17584
P114_740	F	47	3	Verio	0.00296	2.53	1.1	7	0.875	0.96	HeadMatrix	87.5	215	130		0.17584
P114_921	F	48	3	Verio	0.00296	2.53	1.1	7	0.875	0.96	HeadMatrix	100	246	130		0.17584

P114_1106	F	48	3	Verio	0.00296	2.53	1.1	7	0.875	0.96	32Ch_Head	100	246	130		0.17584
P119_0	F	34	3	Verio	0.00296	2.53	1.1	7	0.875	0.96	32Ch_Head	100	246	130		0.17584
P120_0	F	17	3	Verio	0.00296	2.53	1.1	7	0.875	0.96	HeadMatrix	87.5	215	130		0.17584
P120_170	F	18	3	Verio	0.00296	2.53	1.1	7	0.875	0.96	HeadMatrix	87.5	215	130		0.17584
P120_512	F	19	3	Verio	0.00296	2.53	1.1	7	0.875	0.96	HeadMatrix	87.5	215	130		0.17584
P120_694	F	19	3	Verio	0.00296	2.53	1.1	7	0.875	0.96	32Ch_Head	100	246	130		0.17584
P120_876	F	20	3	Verio	0.00296	2.53	1.1	7	0.875	0.96	HeadMatrix	100	246	130		0.17584
P120_1248	F	21	3	Verio	0.00296	2.53	1.1	7	0.875	0.96	HeadMatrix	100	246	130		0.17584
P120_1429	F	21	3	Verio	0.00296	2.53	1.1	7	0.875	0.96	32Ch_Head	100	246	130		0.17584
P122_0	F	33	1.5	Avanto	0.00292	1.83	1.1	15	1	1	32Ch_Head	100	256	130	0.25	0.13807
P122_191	F	34	1.5	Avanto	0.00292	1.83	1.1	15	1	1	32Ch_Head	100	256	130	0.25	0.13807
P122_374	F	34	1.5	Avanto	0.00292	1.83	1.1	15	1	1	32Ch_Head	100	256	130	0.25	0.13807
P122_590	F	35	1.5	Avanto	0.00292	1.83	1.1	15	1	1	32Ch_Head	100	256	130	0.25	0.13807
P122_786	F	35	1.5	Avanto	0.00292	1.83	1.1	15	1	1	32Ch_Head	100	256	130	0.25	0.13807
P125_0	F	38	1.5	Avanto	0.00292	1.83	1.1	15	1	1	32Ch_Head	100	256	130	0.25	0.13807
P125_175	F	39	1.5	Avanto	0.00292	1.83	1.1	15	1	1	32Ch_Head	100	256	130	0.25	0.13807
P125_363	F	39	1.5	Aera	0.00267	1.52	0.9	8	1	1	HeadNeck_20	100	256	150		0.15062
P125_538	F	40	1.5	Aera	0.00267	1.58	0.9	8	1	1	HeadNeck_20	100	256	150		0.15488
P125_742	F	40	1.5	Aera	0.00267	1.58	0.9	8	1	1	HeadNeck_20	100	256	150		0.15488
P125_951	F	41	1.5	Aera	0.00267	1.58	0.9	8	1	1	HeadNeck_20	100	256	150		0.15488
P125_1106	F	41	1.5	Aera	0.00267	1.58	0.9	8	1	1	HeadNeck_20	100	256	150		0.15488
P129_0	M	29	3	Verio	0.00296	2.53	1.1	7	0.875	0.96	HeadMatrix	87.5	215	130		0.17584
P129_224	M	30	3	TrioTim	0.0022	2.53	1.1	9	1	1	HeadMatrix	100	256	199		0.17584
P129_589	M	31	3	Verio	0.00296	2.53	1.1	7	0.875	0.96	HeadMatrix	87.5	215	130		0.17584
P129_947	M	32	3	Verio	0.00296	2.53	1.1	7	0.875	0.96	32Ch_Head	100	246	130		0.17584
P138_0	F	31	1.5	Avanto	0.00292	1.83	1.1	15	1	1	32Ch_Head	100	256	130	0.15	0.13807
P138_181	F	31	1.5	Avanto	0.00292	1.83	1.1	15	1	1	32Ch_Head	100	256	130	0.25	0.13807
P138_359	F	32	1.5	Avanto	0.00292	1.83	1.1	15	1	1	32Ch_Head	100	256	130	0.25	0.13807
P138_498	F	32	1.5	Avanto	0.00292	1.83	1.1	15	1	1	32Ch_Head	100	256	130	0.25	0.13807
P138_728	F	33	1.5	Avanto	0.00292	1.83	1.1	15	1	1	32Ch_Head	100	256	130	0.25	0.13807
P138_908	F	33	1.5	Aera	0.00267	1.58	0.9	8	1	1	Spine_32	100	256	150		0.15488
P139_0	F	42	3	Verio	0.00296	2.53	1.1	7	0.875	0.96	NeckMatrix	87.5	215	130		0.17584
P139_174	F	43	3	Verio	0.00296	2.53	1.1	7	0.875	0.96	HeadMatrix	87.5	215	130		0.17584
P139_346	F	43	3	Verio	0.00296	2.53	1.1	7	0.875	0.96	HeadMatrix	87.5	215	130		0.17584
P139_540	F	44	3	Verio	0.00296	2.53	1.1	7	0.875	0.96	HeadMatrix	100	246	130		0.17584
P139_912	F	45	3	Verio	0.00296	2.53	1.1	7	0.875	0.96	32Ch_Head	100	246	130		0.17584
P142_0	M	42	3	Verio	0.00296	2.53	1.1	7	0.875	0.96	HeadMatrix	87.5	215	130		0.17584
P142_179	M	43	1.5	Aera	0.00267	1.58	0.9	8	1	1	HeadNeck_20	100	256	150		0.15488
P142_354	M	43	1.5	Aera	0.00267	1.58	0.9	8	1	1	HeadNeck_20	100	256	150		0.15488
P142_545	M	44	1.5	Aera	0.00267	1.58	0.9	8	1	1	HeadNeck_20	100	256	150		0.15488
P150_0	M	55	1.5	Avanto	0.00292	1.72	1.1	15	1	1	32Ch_Head	100	256	130	0.25	0.13201
P150_182	M	55	1.5	Avanto	0.00292	1.83	1.1	15	1	1	32Ch_Head	100	256	130	0.25	0.13807
P150_354	M	56	1.5	Avanto	0.00292	1.83	1.1	15	1	1	32Ch_Head	100	256	130	0.25	0.13807
P150_539	M	56	1.5	Avanto	0.00292	1.83	1.1	15	1	1	32Ch_Head	100	256	130	0.25	0.13807
P150_714	M	57	1.5	Avanto	0.00292	1.83	1.1	15	1	1	32Ch_Head	100	256	130	0.25	0.13807
P150_903	M	57	1.5	Avanto	0.00292	1.83	1.1	15	1	1	32Ch_Head	100	256	130	0.25	0.13807
P150_1105	M	58	1.5	Avanto	0.00292	1.83	1.1	15	1	1	32Ch_Head	100	256	130	0.25	0.13807
P155_0	F	39	1.5	Avanto	0.00292	1.83	1.1	15	1	1	32Ch_Head	100	256	130	0.25	0.13807
P155_177	F	39	1.5	Avanto	0.00292	1.83	1.1	15	1	1	32Ch_Head	100	256	130	0.25	0.13807
P155_338	F	40	1.5	Avanto	0.00292	1.83	1.1	15	1	1	32Ch_Head	100	256	130	0.25	0.13807
P155_527	F	40	1.5	Avanto	0.00292	1.83	1.1	15	1	1	32Ch_Head	100	256	130	0.25	0.13807
P155_709	F	41	1.5	Avanto	0.00292	1.72	1.1	15	1	1	32Ch_Head	100	256	130	0.25	0.13201
P155_884	F	41	1.5	Avanto	0.00292	1.83	1.1	15	1	1	32Ch_Head	100	256	130	0.25	0.13807
P156_0	F	44	3	Verio	0.00296	2.53	1.1	7	0.875	0.96	HeadMatrix	87.5	215	130		0.17584
P156_190	F	44	3	Verio	0.00296	2.53	1.1	7	0.875	0.96	HeadMatrix	87.5	215	130		0.17584
P156_371	F	45	3	Verio	0.00296	2.53	1.1	7	0.875	0.96	HeadMatrix	100	246	130		0.17584
P156_553	F	45	3	Verio	0.00296	2.53	1.1	7	0.875	0.96	32Ch_Head	100	246	130		0.17584
P156_734	F	46	1.5	Aera	0.00267	1.58	0.9	8	1	1	HeadNeck_20	100	256	150		0.15488
P156_917	F	46	1.5	Avanto	0.00292	1.83	1.1	15	1	1	HeadMatrix	100	256	130	0.25	0.13807
P157_0	F	25	1.5	Avanto	0.00292	1.83	1.1	15	1	1	32Ch_Head	100	256	130	0.25	0.13807
P157_147	F	25	1.5	Avanto	0.00292	1.83	1.1	15	1	1	32Ch_Head	100	256	130	0.25	0.13807
P157_329	F	26	1.5	Avanto	0.00292	1.83	1.1	15	1	1	32Ch_Head	100	256	130	0.25	0.13807
P157_515	F	26	1.5	Avanto	0.00292	1.72	1.1	15	1	1	32Ch_Head	100	256	130	0.25	0.13201
P157_700	F	27	1.5	Aera	0.00267	1.58	0.9	8	1	1	HeadNeck_20	100	256	150		0.15488
P157_866	F	27	1.5	Avanto	0.00292	1.83	1.1	15	1	1	32Ch_Head	100	256	130	0.25	0.13807
P157_1054	F	28	1.5	Avanto	0.00292	1.83	1.1	15	1	1	32Ch_Head	100	256	130	0.25	0.13807
P158_0	M	29	3	Verio	0.00296	2.53	1.1	7	0.875	0.96	NeckMatrix	87.5	215	130		0.17584
P158_191	M	30	3	Verio	0.00296	2.53	1.1	7	0.875	0.96	HeadMatrix	87.5	215	130		0.17584
P158_370	M	30	3	Verio	0.00296	2.53	1.1	7	0.875	0.96	NeckMatrix	100	246	130		0.17584

P158_561	M	31	3	Verio	0.00296	2.53	1.1	7	0.875	0.96	HeadMatrix	100	246	130		0.17584
P161_0	F	51	3	Verio	0.00296	2.53	1.1	7	0.875	0.96	HeadMatrix	87.5	215	130		0.17584
P161_184	F	52	3	Verio	0.00296	2.53	1.1	7	0.875	0.96	HeadMatrix	87.5	215	130		0.17584
P161_532	F	53	3	Verio	0.00296	2.53	1.1	7	0.875	0.96	32Ch_Head	100	246	130		0.17584
P163_0	M	32	3	Verio	0.00296	2.53	1.1	7	0.875	0.96	NeckMatrix	87.5	215	130		0.17584
P163_185	M	33	3	Verio	0.00296	2.53	1.1	7	0.875	0.96	32Ch_Head	100	246	130		0.17584
P163_370	M	33	3	Verio	0.00296	2.53	1.1	7	0.875	0.96	32Ch_Head	100	246	130		0.17584
P163_551	M	34	3	Verio	0.00296	2.53	1.1	7	0.875	0.96	32Ch_Head	100	246	130		0.17584
P165_0	F	33	1.5	Avanto	0.00292	1.83	1.1	15	1	1	32Ch_Head	100	256	130	0.25	0.13807
P165_97	F	33	1.5	Avanto	0.00292	1.83	1.1	15	1	1	32Ch_Head	100	256	130	0.25	0.13807
P165_249	F	34	1.5	Avanto	0.00292	1.83	1.1	15	1	1	32Ch_Head	100	256	130	0.25	0.13807
P165_422	F	34	1.5	Avanto	0.00292	1.72	1.1	15	1	1	NeckMatrix	100	256	130	0.25	0.13201
P173_0	F	26	1.5	Avanto	0.00292	1.83	1.1	15	1	1	32Ch_Head	100	256	130	0.25	0.13807
P173_175	F	26	1.5	Avanto	0.00292	1.72	1.1	15	1	1	HeadMatrix	100	256	130	0.25	0.13201
P173_567	F	27	3	Verio	0.00296	2.53	1.1	7	0.875	0.96	32Ch_Head	100	246	130		0.17584
P173_738	F	28	1.5	Avanto	0.00292	1.83	1.1	15	1	1	32Ch_Head	100	256	130	0.25	0.13807
P173_911	F	28	1.5	Avanto	0.00292	1.83	1.1	15	1	1	32Ch_Head	100	256	130	0.25	0.13807
P173_1096	F	29	1.5	Avanto	0.00292	1.72	1.1	15	1	1	32Ch_Head	100	256	130	0.25	0.13201
P174_0	M	55	3	Verio	0.00296	2.53	1.1	7	0.875	0.96	HeadMatrix	87.5	215	130		0.17584
P174_174	M	56	3	Verio	0.00296	2.53	1.1	7	0.875	0.96	32Ch_Head	100	246	130		0.17584
P174_273	M	56	3	Verio	0.00296	2.53	1.1	7	0.875	0.96	HeadMatrix	87.5	215	130		0.17584
P174_454	M	56	3	Verio	0.00296	2.53	1.1	7	0.875	0.96	HeadMatrix	100	246	130		0.17584
P174_635	M	57	3	Verio	0.00296	2.53	1.1	7	0.875	0.96	HeadMatrix	100	246	130		0.17584
P174_823	M	57	3	TrioTim	0.0022	2.53	1.1	9	1	1	HeadMatrix	100	256	199		0.17584
P175_0	F	58	1.5	Avanto	0.00292	1.83	1.1	15	1	1	32Ch_Head	100	256	130	0.25	0.13807
P175_182	F	59	1.5	Aera	0.00267	1.58	0.9	8	1	1	HeadNeck_20	100	256	150		0.15488
P175_369	F	59	1.5	Aera	0.00267	1.58	0.9	8	1	1	HeadNeck_20	100	256	150		0.15488
P175_565	F	60	1.5	Aera	0.00267	1.58	0.9	8	1	1	HeadNeck_20	100	256	150		0.15488
P176_263	M	32	1.5	Avanto	0.00292	1.83	1.1	15	1	1	32Ch_Head	100	256	130	0.25	0.13807
P176_0	M	32	1.5	Avanto	0.00292	1.83	1.1	15	1	1	32Ch_Head	100	256	130	0.25	0.13807
P176_178	M	33	1.5	Avanto	0.00292	1.72	1.1	15	1	1	32Ch_Head	100	256	130	0.25	0.13201
P176_354	M	33	1.5	Avanto	0.00292	1.83	1.1	15	1	1	32Ch_Head	100	256	130	0.25	0.13807
P176_509	M	34	1.5	Avanto	0.00292	1.83	1.1	15	1	1	32Ch_Head	100	256	130	0.25	0.13807
P176_696	M	34	1.5	Avanto	0.00292	1.83	1.1	15	1	1	32Ch_Head	100	256	130	0.25	0.13807
P176_872	M	35	1.5	Avanto	0.00292	1.83	1.1	15	1	1	32Ch_Head	100	256	130	0.25	0.13807
P177_0	F	57	1.5	Aera	0.00267	1.58	0.9	8	1	1	Spine_32	100	256	150		0.15488
P177_190	F	58	1.5	Aera	0.00267	1.58	0.9	8	1	1	HeadNeck_20	100	256	150		0.15488
P177_345	F	58	1.5	Aera	0.00267	1.58	0.9	8	1	1	HeadNeck_20	100	256	150		0.15488
P177_551	F	59	1.5	Aera	0.00267	1.58	0.9	8	1	1	HeadNeck_20	100	256	150		0.15488
P177_652	F	59	3	Verio	0.00296	2.53	1.1	7	0.875	0.96	32Ch_Head	100	246	130		0.17584
P183_0	M	31	1.5	Avanto	0.00292	1.83	1.1	15	1	1	32Ch_Head	100	256	130	0.25	0.13807
P183_162	M	31	1.5	Avanto	0.00292	1.83	1.1	15	1	1	32Ch_Head	100	256	130	0.25	0.13807
P183_354	M	32	1.5	Avanto	0.00292	1.83	1.1	15	1	1	32Ch_Head	100	256	130	0.25	0.13807
P183_550	M	32	1.5	Avanto	0.00292	1.83	1.1	15	1	1	32Ch_Head	100	256	130	0.25	0.13807
P183_926	M	33	1.5	Aera	0.00267	1.58	0.9	8	1	1	HeadNeck_20	100	256	150		0.15488
P184_0	M	42	3	Verio	0.00296	2.53	1.1	7	0.875	0.96	HeadMatrix	87.5	215	130		0.17584
P184_177	M	43	3	Verio	0.00296	2.53	1.1	7	0.875	0.96	HeadMatrix	100	246	130		0.17584
P184_359	M	43	1.5	Aera	0.00267	1.58	0.9	8	1	1	HeadNeck_20	100	256	150		0.15488
P184_541	M	44	3	Verio	0.00296	2.53	1.1	7	0.875	0.96	HeadMatrix	100	246	130		0.17584
P184_741	M	44	1.5	Avanto	0.00292	1.83	1.1	15	1	1	NeckMatrix	100	256	130	0.25	0.13807
P184_1149	M	45	1.5	Avanto	0.00292	1.83	1.1	15	1	1	HeadMatrix	100	256	130	0.25	0.13807
P186_0	F	25	1.5	Avanto	0.00292	1.83	1.1	15	1	1	32Ch_Head	100	256	130	0.25	0.13807
P186_141	F	25	1.5	Avanto	0.00292	1.72	1.1	15	1	1	32Ch_Head	100	256	130	0.25	0.13201
P186_415	F	26	1.5	Avanto	0.00292	1.72	1.1	15	1	1	32Ch_Head	100	256	130	0.25	0.13201
P186_632	F	26	1.5	Avanto	0.00292	1.83	1.1	15	1	1	32Ch_Head	100	256	130	0.25	0.13807
P190_0	F	25	1.5	Avanto	0.00292	1.83	1.1	15	1	1	32Ch_Head	100	256	130	0.25	0.13807
P190_107	F	25	1.5	Avanto	0.00292	1.83	1.1	15	1	1	32Ch_Head	100	256	130	0.25	0.13807
P190_434	F	26	1.5	Avanto	0.00292	1.83	1.1	15	1	1	32Ch_Head	100	256	130	0.25	0.13807
P190_757	F	27	1.5	Avanto	0.00292	1.83	1.1	15	1	1	32Ch_Head	100	256	130	0.25	0.13807
P194_0	F	51	1.5	Avanto	0.00292	1.83	1.1	15	1	1	32Ch_Head	100	256	130	0.25	0.13807
P194_176	F	52	1.5	Avanto	0.00292	1.83	1.1	15	1	1	32Ch_Head	100	256	130	0.25	0.13807
P194_938	F	54	1.5	Avanto	0.00292	1.83	1.1	15	1	1	32Ch_Head	100	256	130	0.25	0.13807
P194_1092	F	54	1.5	Avanto	0.00292	1.83	1.1	15	1	1	32Ch_Head	100	256	130	0.25	0.13807
P199_0	F	29	1.5	Aera	0.00267	1.58	0.9	8	1	1	HeadNeck_20	100	256	150		0.15488
P199_73	F	29	1.5	Avanto	0.00292	1.83	1.1	15	1	1	32Ch_Head	100	256	130	0.25	0.13807
P199_262	F	29	1.5	Avanto	0.00292	1.83	1.1	15	1	1	32Ch_Head	100	256	130	0.25	0.13807
P199_437	F	30	1.5	Avanto	0.00292	1.72	1.1	15	1	1	32Ch_Head	100	256	130	0.25	0.13201
P201_0	F	20	3	Verio	0.00296	2.53	1.1	7	0.875	0.96	32Ch_Head	100	246	130		0.17584
P201_183	F	20	3	Verio	0.00296	2.53	1.1	7	0.875	0.96	32Ch_Head	100	246	130		0.17584

P201_364	F	21	3	Verio	0.00296	2.53	1.1	7	0.875	0.96	32Ch_Head	100	246	130		0.17584
P201_583	F	21	3	Verio	0.00296	2.53	1.1	7	0.875	0.96	32Ch_Head	100	246	130		0.17584
P201_778	F	22	3	Verio	0.00296	2.53	1.1	7	0.875	0.96	32Ch_Head	100	246	130		0.17584
P203_0	M	50	3	Verio	0.00296	2.53	1.1	7	0.875	0.96	HeadMatrix	100	246	130		0.17584
P203_330	M	51	3	Verio	0.00296	2.53	1.1	7	0.875	0.96	32Ch_Head	100	246	130		0.17584
P203_514	M	51	3	Verio	0.00296	2.53	1.1	7	0.875	0.96	32Ch_Head	100	246	130		0.17584
P203_694	M	52	3	Verio	0.00296	2.53	1.1	7	0.875	0.96	32Ch_Head	100	246	130		0.17584
P216_0	F	26	3	Verio	0.00296	2.53	1.1	7	0.875	0.96	32Ch_Head	100	246	130		0.17584
P216_214	F	27	3	Verio	0.00296	2.53	1.1	7	0.875	0.96	32Ch_Head	100	246	130		0.17584
P216_397	F	27	3	Verio	0.00296	2.53	1.1	7	0.875	0.96	32Ch_Head	100	246	130		0.17584
P216_581	F	28	3	Verio	0.00296	2.53	1.1	7	0.875	0.96	32Ch_Head	100	246	130		0.17584
P217_0	M	25	3	Verio	0.00296	2.53	1.1	7	0.875	0.96	32Ch_Head	100	246	130		0.17584
P217_175	M	26	3	TrioTim	0.0022	2.53	1.1	9	1	1	HeadMatrix	100	256	199		0.17584
P217_392	M	26	3	Verio	0.00296	2.53	1.1	7	0.875	0.96	32Ch_Head	100	246	130		0.17584
P217_573	M	27	3	Verio	0.00296	2.53	1.1	7	0.875	0.96	32Ch_Head	100	246	130		0.17584
P218_0	F	26	1.5	Avanto	0.00292	1.83	1.1	15	1	1	32Ch_Head	100	256	130	0.25	0.13807
P218_183	F	27	1.5	Avanto	0.00292	1.83	1.1	15	1	1	32Ch_Head	100	256	130	0.25	0.13807
P218_569	F	28	1.5	Avanto	0.00292	1.83	1.1	15	1	1	32Ch_Head	100	256	130	0.25	0.13807
P221_0	M	21	1.5	Aera	0.00267	1.58	0.9	8	1	1	HeadNeck_20	100	256	150		0.15488
P221_169	M	21	1.5	Avanto	0.00292	1.83	1.1	15	1	1	32Ch_Head	100	256	130	0.25	0.13807
P221_469	M	22	1.5	Avanto	0.00292	1.83	1.1	15	1	1	32Ch_Head	100	256	130	0.25	0.13807
P221_693	M	23	1.5	Avanto	0.00292	1.83	1.1	15	1	1	32Ch_Head	100	256	130	0.25	0.13807
P221_968	M	23	1.5	Avanto_fit	0.00292	1.72	1.1	15	1	1	HeadNeck_20	100	256	130	0.25	0.13201
P301_0	M	25	1.5	Avanto_fit	0.00292	1.72	1.1	15	1	1	HeadNeck_20	100	256	130	0.25	0.13201
P302_0	M	31	1.5	Avanto	0.00292	1.83	1.1	15	1	1	32Ch_Head	100	256	130	0.25	0.13807
P302_282	M	32	1.5	Avanto	0.00292	1.83	1.1	15	1	1	32Ch_Head	100	256	130	0.25	0.13807
P302_807	M	33	1.5	Avanto_fit	0.00258	1.79	1.1	15	0.875	1	Spine_32	100	256	170	0.1	0.13587
P302_1205	M	34	1.5	Aera	0.00245	1.75	1.1	8	0.875	1	Spine_32	100	256	170	0.1	0.13366
P303_0	M	27	1.5	Avanto_fit	0.00292	1.72	1.1	15	1	1	HeadNeck_20	100	256	130	0.25	0.13201
P303_191	M	28	1.5	Aera	0.00296	1.73	1.1	15	1	1	HeadNeck_20	100	256	130	0.25	0.13256
P303_282	M	28	1.5	Avanto_fit	0.00258	1.79	1.1	15	0.875	1	HeadNeck_20	100	256	170	0.1	0.13587
P303_379	M	28	1.5	Avanto_fit	0.00258	1.79	1.1	15	0.875	1	HeadNeck_20	100	256	170	0.1	0.13587
P303_730	M	29	1.5	Avanto_fit	0.00258	1.79	1.1	15	0.875	1	HeadNeck_20	100	256	170	0.1	0.13587
P306_0	M	29	1.5	Avanto	0.00292	1.83	1.1	15	1	1	32Ch_Head	100	256	130	0.25	0.13807
P306_175	M	32	1.5	Avanto_fit	0.00258	1.79	1.1	15	0.875	1	HeadNeck_20	100	256	170	0.1	0.13587
P306_219	M	30	1.5	Avanto	0.00292	1.83	1.1	15	1	1	32Ch_Head	100	256	130	0.25	0.13807
P306_425	M	30	1.5	Avanto_fit	0.00292	1.72	1.1	15	1	1	HeadNeck_20	100	256	130	0.25	0.13201
P306_595	M	31	1.5	Avanto_fit	0.00241	1.69	1.1	8	0.875	1	HeadNeck_20	100	256	170	0.1	0.13036
P306_825	M	31	1.5	Avanto_fit	0.00258	1.79	1.1	15	0.875	1	HeadNeck_20	100	256	170	0.1	0.13587
P306_1056	M	32	1.5	Avanto_fit	0.00258	1.79	1.1	15	0.875	1	HeadNeck_20	100	256	170	0.1	0.13587
P308_0	F	43	3	Verio	0.00296	2.53	1.1	7	0.875	0.96	32Ch_Head	100	246	130		0.17584
P308_153	F	43	3	Skyra_fit	0.00226	2.07	1.1	8	0.875	1	HeadNeck_64	100	256	200	0.1	0.15126
P308_264	F	43	3	Prisma_fit	0.00226	1.71	1.1	8	0.875	1	HeadNeck_64	100	256	200		0.13146
P308_364	F	44	3	Prisma_fit	0.00226	1.71	1.1	8	0.875	1	HeadNeck_64	100	256	200		0.13146
P309_0	F	53	3	Verio	0.00296	2.53	1.1	7	0.875	0.96	HeadMatrix	87.5	215	130		0.17584
P309_196	F	53	3	Verio	0.00296	2.53	1.1	7	0.875	0.96	32Ch_Head	87.5	215	130		0.17584
P309_378	F	54	3	Verio	0.00296	2.53	1.1	7	0.875	0.96	32Ch_Head	100	246	130		0.17584
P309_742	F	55	3	Verio	0.00296	2.53	1.1	7	0.875	0.96	32Ch_Head	100	246	130		0.17584
P309_933	F	55	3	Verio	0.00296	2.53	1.1	7	0.875	0.96	32Ch_Head	100	246	130		0.17584
P309_1115	F	56	3	Verio	0.00296	2.53	1.1	7	0.875	0.96	32Ch_Head	100	246	130		0.17584
P309_1296	F	56	3	Verio	0.00296	2.53	1.1	7	0.875	0.96	32Ch_Head	100	246	130		0.17584
P314_0	F	43	3	Verio	0.00296	2.53	1.1	7	0.875	0.96	32Ch_Head	100	246	130		0.17584
P314_302	F	44	3	Verio	0.00296	2.53	1.1	7	0.875	0.96	32Ch_Head	100	246	130		0.17584
P314_468	F	44	3	Vida	0.00226	1.8	1.1	8				100	256	199		0.13642
P318_0	F	31	1.5	Avanto	0.00292	1.83	1.1	15	1	1	32Ch_Head	100	256	130	0.25	0.13807
P318_223	F	32	1.5	Avanto	0.00292	1.83	1.1	15	1	1	32Ch_Head	100	256	130	0.25	0.13807
P318_391	F	32	1.5	Avanto	0.00292	1.83	1.1	15	1	1	32Ch_Head	100	256	130	0.25	0.13807
P318_625	F	33	1.5	Avanto	0.00292	1.83	1.1	15	1	1	32Ch_Head	100	256	130	0.25	0.13807
P319_0	F	27	3	Verio	0.00296	2.53	1.1	7	0.875	0.96	HeadMatrix	87.5	215	130		0.17584
P322_0	F	30	1.5	Avanto	0.00292	1.83	1.1	15	1	1	32Ch_Head	100	256	130	0.25	0.13807
P322_209	F	30	1.5	Avanto	0.00292	1.72	1.1	15	1	1	NeckMatrix	100	256	130	0.25	0.13201
P322_363	F	31	1.5	Avanto	0.00292	1.83	1.1	15	1	1	32Ch_Head	100	256	130	0.25	0.13807
P322_462	F	31	1.5	Avanto	0.00292	1.83	1.1	15	1	1	32Ch_Head	100	256	130	0.25	0.13807
P322_771	F	32	1.5	Avanto	0.00292	1.83	1.1	15	1	1	32Ch_Head	100	256	130	0.25	0.13807
P322_888	F	32	1.5	Avanto	0.00292	1.83	1.1	15	1	1	32Ch_Head	100	256	130	0.25	0.13807
P322_1271	F	33	1.5	Avanto	0.00292	1.83	1.1	15	1	1	32Ch_Head	100	256	130	0.25	0.13807
P326_0	F	17	3	Verio	0.00298	2.3	1.1	9	1	1	32Ch_Head	100	256	238		0.16371
P326_175	F	18	3	Verio	0.00296	2.53	1.1	7	0.875	0.96	32Ch_Head	100	246	130		0.17584
P401_0	F	50	1.5	Avanto_fit	0.00292	1.72	1.1	15	1	1	Spine_32	100	256	130	0.25	0.13201

P401_399	F	51	3	Skyra_fit	0.00226	2.07	1.1	8	0.875	1	HeadNeck_64	100	256	200	0.1	0.15126
P401_910	F	53	3	Skyra_fit	0.00226	2.07	1.1	8	0.875	1	HeadNeck_20	100	256	200	0.1	0.15126
P402_0	M	32	1.5	Avanto	0.00292	1.83	1.1	15	1	1	32Ch_Head	100	256	130	0.25	0.13807
P402_60	M	33	1.5	Avanto	0.00292	1.83	1.1	15	1	1	32Ch_Head	100	256	130	0.25	0.13807
P402_249	M	33	1.5	Avanto_fit	0.00241	1.69	1.1	8	0.875	1	HeadNeck_20	100	256	170	0.1	0.13036
P402_424	M	33	1.5	Avanto_fit	0.00241	1.69	1.1	8	0.875	1	HeadNeck_20	100	256	170	0.1	0.13036
P402_438	M	34	3	Skyra_fit	0.00226	2.07	1.1	8	0.875	1	HeadNeck_64	100	256	200	0.1	0.15126
P403_0	F	17	1.5	Aera	0.00267	1.58	0.9	8	1	1	HeadNeck_20	100	256	150		0.15488
P403_254	F	18	1.5	Avanto_fit	0.00258	1.79	1.1	15	0.875	1	HeadNeck_20	100	256	170	0.1	0.13587
P403_433	F	19	1.5	Avanto_fit	0.00258	1.79	1.1	15	0.875	1	HeadNeck_20	100	256	170	0.1	0.13587
P403_652	F	19	1.5	Aera	0.00296	1.73	1.1	15	1	1	Spine_32	100	256	130	0.25	0.13256
P403_833	F	20	1.5	Aera	0.00245	1.75	1.1	8	0.875	1	Spine_32	100	256	170	0.1	0.13366
P403_1016	F	20	1.5	Avanto_fit	0.00258	1.79	1.1	15	0.875	1	HeadNeck_20	100	256	170	0.1	0.13587

2 MS study using original T1 scans

2.1 Statistics for our original model

```
                 Mixed Linear Model Regression Results
========================================================================
Model:            MixedLM Dependent Variable: origMeanThicknessResidual
No. Observations: 285     Method:             ML
No. Groups:       59      Scale:              0.0017
Min. group size:  1       Log-Likelihood:     403.4161
Max. group size:  7       Converged:          Yes
Mean group size:  4.8
-------------------------------------------------------------------------
                         Coef.  Std.Err.    z    P>|z|  [0.025  0.975]
-------------------------------------------------------------------------
Intercept                 0.324    0.112   2.905 0.004   0.105   0.543
Sex[T.m]                 -0.034    0.030  -1.124 0.261  -0.092   0.025
SCANNER_ID[T.MR 3]       -0.065    0.015  -4.264 0.000  -0.095  -0.035
SCANNER_ID[T.MR 4]        0.038    0.053   0.709 0.478  -0.067   0.142
SCANNER_ID[T.MR 5]        0.022    0.055   0.401 0.689  -0.086   0.130
SCANNER_ID[T.MR 5.1]      0.089    0.069   1.287 0.198  -0.047   0.225
SCANNER_ID[T.MR 6]        0.079    0.059   1.344 0.179  -0.036   0.194
SCANNER_ID[T.MR 6.1]      0.011    0.062   0.178 0.859  -0.111   0.133
SCANNER_ID[T.OPMR]       -0.035    0.029  -1.207 0.227  -0.091   0.022
fieldStrength            -0.052    0.031  -1.686 0.092  -0.113   0.008
synth_contrast           -2.155    0.713  -3.022 0.003  -3.552  -0.757
EDSS                      0.002    0.005   0.353 0.724  -0.008   0.012
Group Var                 0.011    0.059
========================================================================
```

2.2 Statistics for our finetuned model

```
                 Mixed Linear Model Regression Results
========================================================================
Model:            MixedLM Dependent Variable: origMeanThicknessResidual
No. Observations: 285     Method:             ML
No. Groups:       59      Scale:              0.0006
Min. group size:  1       Log-Likelihood:     533.3189
Max. group size:  7       Converged:          Yes
Mean group size:  4.8
-------------------------------------------------------------------------
                         Coef.  Std.Err.    z    P>|z|  [0.025  0.975]
-------------------------------------------------------------------------
Intercept                 0.073    0.068   1.072 0.284  -0.060   0.206
Sex[T.m]                 -0.026    0.023  -1.126 0.260  -0.071   0.019
SCANNER_ID[T.MR 3]       -0.056    0.009  -6.086 0.000  -0.074  -0.038
SCANNER_ID[T.MR 4]        0.017    0.032   0.541 0.588  -0.046   0.080
SCANNER_ID[T.MR 5]        0.003    0.033   0.076 0.939  -0.062   0.067
SCANNER_ID[T.MR 5.1]      0.058    0.042   1.388 0.165  -0.024   0.140
SCANNER_ID[T.MR 6]        0.020    0.036   0.553 0.580  -0.050   0.089
SCANNER_ID[T.MR 6.1]      0.036    0.038   0.970 0.332  -0.037   0.110
SCANNER_ID[T.OPMR]       -0.046    0.017  -2.664 0.008  -0.080  -0.012
fieldStrength            -0.035    0.019  -1.899 0.058  -0.072   0.001
synth_contrast           -0.603    0.431  -1.398 0.162  -1.448   0.242
EDSS                      0.005    0.003   1.555 0.120  -0.001   0.012
Group Var                 0.007    0.059
========================================================================
```

3 MS study with lesion filled scans

3.1 Lesion filling results

Figure 1 shows an example of T1 scan, the registered lesion mask, and the lesion-filling resulting T1 for a random subject.

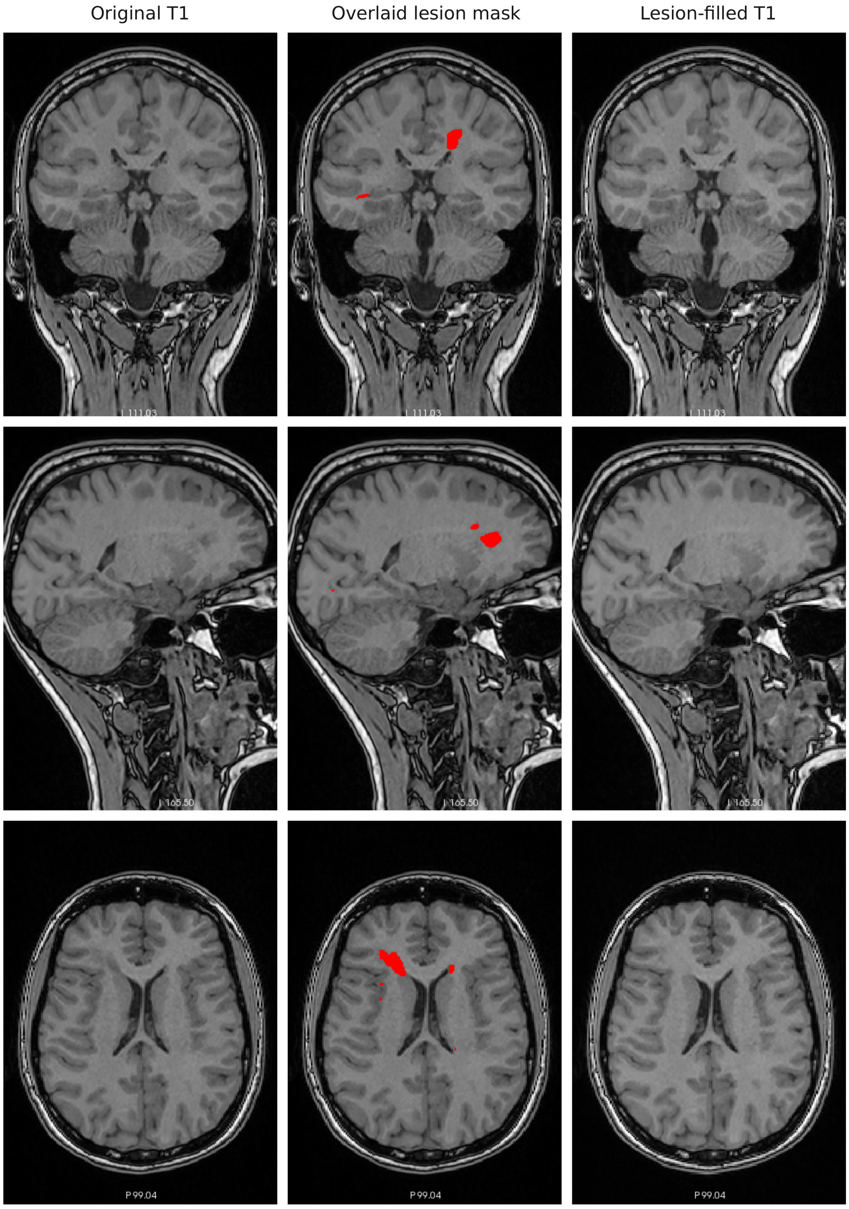

Figure 1: Original T1, lesion mask, and resulting lesion-filled scan for a random MS subject. Lesion masks were obtained by registering Nabla lesion masks [1] from same session FLAIR scans to T1 space. Lesion filling was performed using [2].

3.2 Statistics for our finetuned model

```
                 Mixed Linear Model Regression Results
========================================================================
Model:             MixedLM Dependent Variable: origMeanThicknessResidual
No. Observations:  283     Method:             REML
No. Groups:        59      Scale:              0.0007
Min. group size:   1       Log-Likelihood:     487.6187
Max. group size:   7       Converged:          Yes
```

Mean group size: 4.8

	Coef.	Std.Err.	z	P>|z|	[0.025	0.975]
Intercept	0.070	0.071	0.977	0.328	-0.070	0.210
Sex[T.m]	-0.026	0.023	-1.144	0.253	-0.070	0.019
SCANNER_ID[T.MR 3]	-0.059	0.010	-6.156	0.000	-0.078	-0.041
SCANNER_ID[T.MR 4]	0.024	0.034	0.719	0.472	-0.042	0.090
SCANNER_ID[T.MR 5]	0.009	0.035	0.249	0.803	-0.059	0.077
SCANNER_ID[T.MR 5.1]	0.068	0.044	1.554	0.120	-0.018	0.154
SCANNER_ID[T.MR 6]	0.028	0.037	0.746	0.456	-0.045	0.101
SCANNER_ID[T.MR 6.1]	0.044	0.039	1.121	0.262	-0.033	0.122
SCANNER_ID[T.OPMR]	-0.053	0.018	-2.946	0.003	-0.089	-0.018
fieldStrength	-0.039	0.020	-1.996	0.046	-0.077	-0.001
synth_contrast	-0.515	0.455	-1.132	0.258	-1.406	0.377
EDSS	0.005	0.003	1.532	0.126	-0.001	0.012
Group Var	0.006	0.055				

3.3 Influence of lesion filling on regional maps of $\beta_{\mathbf{contrast}}$

Figure 2 shows regional maps of β_{contrast} for our original segmentation model applied on original scans, the finetuned model applied to original T1 scans, and the finetuned model applied to lesion-filled scans. While 12 regions show significant effect of contrast on the residual thickness (labeled with red asterisks on Figure 2), none is significant when using the finetuned model on either the original T1 scans or the lesion-filled scans.

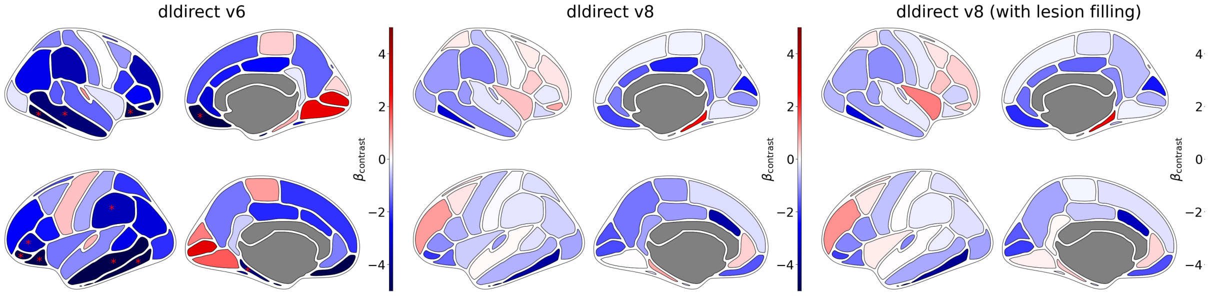

Figure 2: Regional map of β_{contrast} derived from the original model applied on original T1 scans (dldirect v1), the finetuned model applied to original T1 scans (dldirect v8), and the finetuned model applied to lesion-filled scans (dldirect v8 with lesion-filling). Regions with significant effect of β_{contrast} after Bonferroni correction ($p < 0.000735$) are labeled with a red asterix.

3.4 Deep gray matter volume association with contrast

To study the association of subcortical region volume with contrast, we performed the same mixed effects analyzes described for thickness in section 2.4.3 of the main text, but for the volume estimates of the thalamus proper, pallidum, caudate, putamen and hippocampus and amygdala.

3.4.1 Original model v6

	Coef.	Std.Err.	z	P>|z|	[0.025	0.975]
Region						
Left Thalamus Proper	-8656.652222	2122.177213	-4.079137	0.000542	-12816.043127	-4497.261317
Left Caudate	2016.115948	1237.787289	1.628806	1.000000	-409.902559	4442.134454
Left Putamen	8799.458244	1924.211414	4.573021	0.000058	5028.073174	12570.843315
Left Pallidum	2738.835650	1205.673446	2.271623	0.277311	375.759118	5101.912182
Left Hippocampus	-722.106391	1497.019969	-0.482363	1.000000	-3656.211614	2211.998833
Left Amygdala	-15.563869	1274.811694	-0.012209	1.000000	-2514.148877	2483.021139
Right Thalamus Proper	-7230.758020	2372.119865	-3.048226	0.027624	-11880.027523	-2581.488517
Right Caudate	2753.815317	1443.913587	1.907188	0.677954	-76.203311	5583.833944
Right Putamen	7060.060479	1866.863466	3.781777	0.001869	3401.075322	10719.045637
Right Pallidum	738.902926	1194.882370	0.618390	1.000000	-1603.023484	3080.829337
Right Hippocampus	1385.482970	2098.963580	0.660080	1.000000	-2728.410051	5499.375992
Right Amygdala	-1518.204099	1122.485432	-1.352538	1.000000	-3718.235118	681.826920

3.4.2 Fine-tuned v8

	Coef.	Std.Err.	z	P>|z|	[0.025	0.975]
Region						
Left Thalamus Proper	-2881.314497	1982.717944	-1.453215	1.000000	-6767.370259	1004.741264
Left Caudate	5227.839127	1654.233325	3.160279	0.018914	1985.601387	8470.076867
Left Putamen	4669.755821	1844.232918	2.532086	0.136064	1055.125723	8284.385919
Left Pallidum	1216.425410	1150.173003	1.057602	1.000000	-1037.872252	3470.723072
Left Hippocampus	1075.735883	1613.557002	0.666686	1.000000	-2086.777727	4238.249494
Left Amygdala	2275.505683	1386.579991	1.641092	1.000000	-442.141162	4993.152528
Right Thalamus Proper	-5591.864056	2400.942245	-2.329029	0.238290	-10297.624385	-886.103727
Right Caudate	4742.984139	1581.067297	2.999862	0.032412	1644.149180	7841.819098
Right Putamen	4262.017824	1958.800299	2.175831	0.354815	422.839785	8101.195863
Right Pallidum	-272.697799	1117.467224	-0.244032	1.000000	-2462.893313	1917.497714
Right Hippocampus	2535.103500	1573.467350	1.611157	1.000000	-548.835836	5619.042837
Right Amygdala	1748.303420	1134.104284	1.541572	1.000000	-474.500130	3971.106971

References

[1] McKinley, R., Wepfer, R., Gundersen, T., Wagner, F., Chan, A., Wiest, R., and Reyes, M. Nabla-net: A deep dag-like convolutional architecture for biomedical image segmentation. In *International Workshop on Brainlesion: Glioma, Multiple Sclerosis, Stroke and Traumatic Brain Injuries* (2016), Springer, pp. 119–128.

[2] Uhr, V., Diaz, I., Rummel, C., and McKinley, R. Exploring robustness of cortical morphometry in the presence of white matter lesions, using diffusion models for lesion filling, 2025.
